# Comparisons between wrinkles and photo-ageing detected and self-reported by the participant or identified by trained assessors reveal insights from Chinese individuals in the Singapore/Malaysia Cross-sectional Genetics Epidemiology Study (SMCGES) cohort

**DOI:** 10.1186/s40101-024-00361-8

**Published:** 2024-05-18

**Authors:** Jun Yan Ng, Hongyu Zhou, Tianqi Li, Fook Tim Chew

**Affiliations:** 1 Department of Biological Sciences, Faculty of Science, National University of Singapore, Singapore, 117543 Singapore; 2 Allergy and Molecular Immunology Laboratory, Lee Hiok Kwee Functional Genomics Laboratories, Block S2, Level 5, 14 Science Drive 4, Lower Kent Ridge Road, Singapore, 117543 Singapore

## Abstract

**Background:**

Changes develop on the facial skin as a person ages. Other than chronological time, it has been discovered that gender, ethnicity, air pollution, smoking, nutrition, and sun exposure are notable risk factors that influence the development of skin ageing phenotypes such as wrinkles and photo-ageing. These risk factors can be quantified through epidemiological collection methods. We previously studied wrinkles and photo-ageing in detail using photo-numeric scales. The analysis was performed on the ethnic Chinese skin by three trained assessors. Recent studies have shown that it is possible to use self-reported data to identify skin-related changes including skin colour and skin cancer. In order to investigate the association between risk factors and skin ageing phenotypic outcomes in large-scale epidemiological studies, it would be useful to evaluate whether it is also possible for participants to self-report signs of ageing on their skin.

**Aim:**

We have previously identified several validated photo-numeric scales for wrinkling and photo-ageing to use on ethnic Chinese skin. Using these scales, our trained assessors grade wrinkling and photo-ageing with moderately high inter-assessor concordance and agreement. The main objective of this study involves letting participants grade self-reported wrinkling and photo-ageing using these same scales. We aim to compare the concordance and agreement between signs of skin ageing by the participant and signs of ageing identified by our assessors.

**Method:**

Three trained assessors studied facial photo-ageing on 1081 ethnic Chinese young adults from the Singapore/Malaysia Cross-sectional Genetics Epidemiology Study (SMCGES) cohort. Self-reported facial photo-ageing data by the same 1081 participants were also collated and the two sets of data are compared.

**Results:**

Here, we found that self-reported signs of photo-ageing are concordant with photo-ageing detected by our assessors. This finding is consistent whether photo-ageing is evaluated through studying wrinkle variations (Spearman’s rank correlation (ρ) value: 0.246–0.329) or through studying dyspigmentation patterns (Spearman’s rank correlation (ρ) value 0.203–0.278). When studying individual wrinkles, both participants and assessors often detect the presence of the same wrinkle (Spearman’s rank correlation (ρ) value 0.249–0.366). A weak-to-fair level of agreement between both participants and assessors (Cohen’s kappa (κ) values: 0.041–0.233) persists and is statistically significant after accounting for agreements due to chance. Both the participant and the assessor are largely consistent in evaluating the extent of photo-ageing (area under curve (AUC) values 0.689–0.769) and in discerning between the presence or absence of a given facial wrinkle (area under curve (AUC) values 0.601–0.856).

**Conclusion:**

When we analyse the overall appearance of the face, our results show that signs of photo-ageing identified by the participant are concordant with signs of photo-ageing identified by our assessors. When we focused our analysis on specific areas of the face, we found that participants were more likely to identify and self-report the same wrinkles that our assessors have also detected. Here, we found that self-reported signs of skin ageing provide a satisfactory approximation to the signs of skin ageing identified by our assessors. The ability to use self-reported signs of skin ageing should also be evaluated on scales beyond the ones discussed in this study. Currently, there are not as many photo-numeric scales for quantifying dyspigmentation patterns as there are for quantifying wrinkle variations. As Chinese skin is known to become dyspigmented more easily with age, more photo-numeric scales need to be developed and properly validated.

**Supplementary Information:**

The online version contains supplementary material available at 10.1186/s40101-024-00361-8.

## Introduction

Skin ageing can take many forms. We have previously discussed wrinkles (Ng JY, & Chew FT. Comparisons between Caucasian validated photo-numeric scales and Chinese validated photo-numeric scales for assessing facial wrinkles: Crow’s Feet wrinkles, forehead wrinkles, glabellar frowns, and nasolabial folds. Insights from the Singapore/Malaysia Cross-sectional Genetics Epidemiology Study (SMCGES) cohort. Manuscript submitted for publication in Scientific Reports) and photo-ageing (Ng JY, & Chew FT. Comparisons between Caucasian validated photo-numeric scales and Korean validated photo-numeric scales for photo-ageing. Insights from the Singapore/Malaysia Cross-sectional Genetics Epidemiology Study (SMCGES) cohort. Manuscript submitted for publication in Skin Research and Technology) in detail. In our two earlier studies, we identified validated photo-numeric scales suitable to be used on the ethnic Chinese. Using these scales, all three of our trained assessors consistently grade signs of ageing on the skin in similar ways.

We recently published a meta-analysis [23] where we reviewed that age, gender, ethnicity, air pollution, smoking, nutrition, and sun exposure are notable risk factors that influence the development of skin ageing phenotypes such as wrinkles and photo-ageing. Meanwhile, recent studies have found that it is possible to use self-reported data to identify skin-related changes such as skin colour [15] and skin cancer [3, 4].

We wonder if the methodology of using self-reported data to report skin characteristics can be extended to skin ageing phenotypes. This is because using self-reported skin ageing data can potentially improve the scalability of our findings in large-scale epidemiological studies. This can in turn allow us to explore the associations between risk factors and different forms of skin ageing in a larger population. Therefore, the main objective of this study is to examine how the photo-numeric scales perform using self-reported data as compared to using data evaluated by our trained assessors. Using the results of these comparisons, we seek to evaluate whether self-reported signs of skin ageing provide a satisfactory approximation to the signs of skin ageing identified by our assessors.

## Methods

### Participant recruitment

We recruited from the Singapore/Malaysia Cross-section Genetics Epidemiology Study (SMCGES) cohort for this study. The SMCGES cohort consists of participants from Singapore and Malaysia and has been previously studied in epidemiological and genetic studies of allergic diseases. We have previously described this cohort in detail elsewhere [12, 24, 25].

Using emails and posters, we recruited participants for the previous study (i.e., the epidemiological and genetic study) from the National University of Singapore, Singapore (2005 to 2023), Universiti Tunku Abdul Rahman (UTAR) Campus, Malaysia (2016 to 2018), and Sunway University, Malaysia (2019 and 2022). These participants were recruited through walk-ins and participated in our study voluntarily.

Some participants consent to be re-contacted. We invited these participants to participate in this present study (i.e., the skin ageing study). A total of 10,248 participants were invited to participate in the skin ageing study, of which 3365 completed the study. The demographics of the respondents and non-respondents are similar and can be found elsewhere (Ng JY, & Chew FT. Comparisons between Caucasian validated photo-numeric scales and Chinese validated photo-numeric scales for assessing facial wrinkles: Crow’s Feet wrinkles, forehead wrinkles, glabellar frowns, and nasolabial folds. Insights from the Singapore/Malaysia Cross-sectional Genetics Epidemiology Study (SMCGES) cohort. Manuscript submitted for publication in Scientific Reports) (Ng JY, & Chew FT. Comparisons between Caucasian validated photo-numeric scales and Korean validated photo-numeric scales for photo-ageing. Insights from the Singapore/Malaysia Cross-sectional Genetics Epidemiology Study (SMCGES) cohort. Manuscript submitted for publication in Skin Research and Technology). All 3365 participants constitute our study population. From this study population, we randomly selected a subset of 1081 ethnic Chinese young adult participants (Table 1). This subset has been previously studied for their facial wrinkles (Crow’s Feet wrinkles, forehead wrinkles, glabellar frowns, and nasolabial folds) (Ng JY, & Chew FT. Comparisons between Caucasian validated photo-numeric scales and Chinese validated photo-numeric scales for assessing facial wrinkles: Crow’s Feet wrinkles, forehead wrinkles, glabellar frowns, and nasolabial folds. Insights from the Singapore/Malaysia Cross-sectional Genetics Epidemiology Study (SMCGES) cohort. Manuscript submitted for publication in Scientific Reports) and photo-ageing (Ng JY, & Chew FT. Comparisons between Caucasian validated photo-numeric scales and Korean validated photo-numeric scales for photo-ageing. Insights from the Singapore/Malaysia Cross-sectional Genetics Epidemiology Study (SMCGES) cohort. Manuscript submitted for publication in Skin Research and Technology).

**Table 1:** Summary table for demographics^a^ drawn from a population of young Singapore ethnic Chinese adults recruited from the Singapore/Malaysia Cross-section Genetics Epidemiology Study (SMCGES) cohort

Demographic factor	Subset evaluated	All participants
Participants	1081 (100%)	3365 (100%)
Mean age (years) ± SD	26.15 ± 8.11	26.30 ± 6.92
Mean height (cm) ± SD	166.03 ± 8.60	165.37 ± 8.69
Mean weight (kg) ± SD	60.96 ± 12.60	60.86 ± 13.27
BMI (kg/m^2^) ± SD	22.02 ± 3.99	22.19 ± 4.93
Male	420 (38.85%)	1227 (36.46%)
Female	661 (61.15%)	2138 (63.54%)
Chinese	1081 (100%)	2885 (85.74%)
Low	117 (10.82%)	386 (11.47%)
Moderate	275 (25.44%)	774 (23.00%)
High	226 (20.91%)	792 (23.54%)
Very high	446 (41.26%)	1396 (41.49%)
Missing/invalid	17 (1.57%)	17 (0.51%)
Public housing	664 (61.42%)	1794 (53.31%)
Condominium/private apartment	320 (29.60%)	852 (25.32%)
Landed property	76 (7.03%)	698 (20.74%)
Missing/invalid	21 (1.94%)	21 (0.62%)

*Abbreviations*: *SD* standard deviation, *SGD* Singapore dollars, *HDB (public housing)* Public housing constructed by the Singapore Housing Development Board, *BMI* body mass index, cm, centimetres, *m*^*2*^ squared metres, *kg* kilograms

^a^The values after ± are standard deviation values. Missing/Invalid refers to responses that are either left blank or otherwise invalid

The study was conducted in accordance with the Declaration of Helsinki and Good Clinical Practices.

### Survey data collection

The SMCGES cohort participants have previously completed a set of investigator-administered, validated International Study of Asthma and Allergies in Childhood (ISAAC) questionnaires. These questionnaires collated sociodemographic data, personal lifestyle data, and familial and personal medical history data.

After completing the ISAAC questionnaires, most of the participants agreed to take part in a separate study on skin ageing (i.e. this present study). All these participants signed an informed consent form to participate in the skin ageing study which contains an investigator-administered skin ageing questionnaire. In this questionnaire, participants self-reported various skin ageing phenotypes on their skin; this will be elaborated on in the “Evaluating skin ageing” section. After completing the self-evaluation, participants completed more personal lifestyle questions on the questionnaire.

Further details on the consent process can be obtained from the “Ethics approval and consent to participate” section.

### Image data collection

Investigators acquire photographic documentation of all participants. Photographs are taken with the same camera (Canon EOS 6DII Body with an EF85 f/1.4L IS USM lens) and tripod positioned one metre away from the participant. Photographs taken meet the standards of other recent and similar studies [2, 6]. In brief, photographs are taken with identical camera settings, lighting, and positioning at five angles–*en face*, 45° oblique, and 90° side profiles. A total of 5 photos are collected from each participant.

### Evaluating skin ageing

Photo-ageing is evaluated by (a) the participant, and (b) three trained assessors on multiple validated photo-numeric scales reported in Table S1. The same photo-numeric scales are used by all participants and assessors.

A dressing table mirror was provided for each participant. With the aid of the mirror, each participant independently evaluates different signs of ageing on their skin. These signs of skin ageing include wrinkling phenotypes (Crow’s Feet wrinkles, forehead wrinkles, glabellar frowns, and nasolabial folds) and photo-ageing phenotypes. Other skin ageing phenotypes are reported in the supplementary materials (Tables S1 and S2).

The three trained assessors evaluate skin ageing phenotypes from photographs and the assessment scores are calibrated before use. The calibration process has been described previously (Ng JY, & Chew FT. Comparisons between Caucasian validated photo-numeric scales and Chinese validated photo-numeric scales for assessing facial wrinkles: Crow’s Feet wrinkles, forehead wrinkles, glabellar frowns, and nasolabial folds. Insights from the Singapore/Malaysia Cross-sectional Genetics Epidemiology Study (SMCGES) cohort. Manuscript submitted for publication in Scientific Reports) (Ng JY, & Chew FT. Comparisons between Caucasian validated photo-numeric scales and Korean validated photo-numeric scales for photo-ageing. Insights from the Singapore/Malaysia Cross-sectional Genetics Epidemiology Study (SMCGES) cohort. Manuscript submitted for publication in Skin Research and Technology). Briefly, 30 participants were randomly selected, their photographs were openly discussed, and a consensus among all three assessors was reached. Except for this small handful of participants (*n* = 30), all the 1081 participants are assessed three times independently.

### Statistical analysis

All the photo-numeric scales are standardised.

Two-tailed bivariate correlations for Spearman’s rank correlation (ρ) are calculated using Version 25 of the IBM Statistical Package for Social Scientists (SPSS/PC) and reported in Tables 2 and S2. Qualitative interpretations of the Spearman’s Rank correlation (ρ) values follow the naming practices for the strength of correlation coefficients used in healthcare and related fields [1, 5]. The strengths are interpreted as follows: 0.00: no correlation, 0.01–0.20: weak, 0.21–0.50: fair, 0.51–0.70: moderate, 0.71–0.90: very strong, 0.91–1.00: perfect.

**Table 2:** Comparison between skin ageing phenotypes grades by assessors and participants evaluated using Spearman correlation, Cohen's kappa, and area under the receiver operating characteristic (ROC) curve (AUC)

Category	Phenotype	Test	The self-reported phenotype by the participant is compared against:					
			An investigator-administered grading by our assessors (lax definition^a^)		An investigator-administered grading by our assessors (moderately-strict definition^b^)		An investigator-administered grading by our assessors (strict definition^c^)	
			Value	*p* value	Value	*p* value	Value	*p* value
Wrinkles	Crow’s Feet wrinkles	Spearman’s rank correlation (ρ)	0.331	4.65 × 10^−29^	0.281	4.64 × 10^−21^	0.346	1.04 × 10^−31^
		Cohen’s kappa (κ)	0.101	9.98 × 10^−16^	0.180	4.42 × 10^−15^	0.109	1.00 × 10^−11^
		Area under curve (AUC) (assessor)^d^	0.671	1.79 × 10^−11^	0.798	5.28 × 10^−15^	0.772	1.09 × 10^−11^
		Area under curve (AUC) (participant)^e^	0.722	1.45 × 10^**−**13^	0.659	1.16 × 10^**−**7^	0.731	1.34 × 10^**−**14^
Wrinkles	Forehead wrinkles	Spearman’s rank correlation (ρ)	0.286	3.64 × 10^**−**32^	0.348	4.34 × 10^**−**32^	0.307	4.90 × 10^**−**25^
		Cohen’s kappa (κ)	0.134	6.71 × 10^**−**20^	0.233	5.79 × 10^**−**25^	0.105	2.75 × 10^**−**12^
		Area under curve (AUC) (assessor) ^d^	0.671	3.41 × 10^**−**11^	0.828	7.36 × 10^**−**18^	0.765	2.60 × 10^**−**13^
		Area under curve (AUC) (participant)^e^	0.692	1.60 × 10^−12^	0.686	7.01 × 10^−12^	0.704	5.66 × 10^−14^
Wrinkles	Glabellar frowns	Spearman’s rank correlation (ρ)	0.273	5.42 × 10^**−**20^	0.289	2.92 × 10^**−**22^	0.249	9.85 × 10^**−**17^
		Cohen’s kappa (κ)	0.170	5.74 × 10^**−**17^	0.113	6.98 × 10^**−**16^	0.065	6.27 × 10^**−**13^
		Area under curve (AUC) (assessor) ^d^	0.795	8.70 × 10^**−**5^	0.911	1.21 × 10^**−**7^	0.840	5.10 × 10^**−**5^
		Area under curve (AUC) (participant)^e^	0.615	2.09 × 10^**−**3^	0.614	2.30 × 10^**−**3^	0.601	6.85 × 10^**−**3^
Wrinkles	Nasolabial folds	Spearman’s rank correlation (ρ)	0.339	1.55 × 10^**−**30^	0.351	1.25 × 10^**−**32^	0.366	1.42 × 10^35^
		Cohen’s kappa (κ)	0.053	9.65 × 10^−4^	0.190	2.58 × 10^−25^	0.136	2.07 × 10^−13^
		Area under curve (AUC) (assessor) ^d^	0.771	3.17 × 10^−14^	0.847	7.40 × 10^−16^	0.771	3.17 × 10^−14^
		Area under curve (AUC) (participant)^e^	0.839	3.49 × 10^−9^	0.877	4.75 × 10^−11^	0.856	5.34 × 10^−10^
Photo-ageing	Wrinkling constituent of photo-ageing as measured by the Larnier scale	Spearman’s rank correlation (ρ)	0.246	2.24 × 10^**−**16^	0.383	4.86 × 10^**−**39^	0.329	1.09 × 10^−28^
		Cohen’s kappa (κ)	0.041	9.80 × 10^**−**5^	0.149	5.41 × 10^**−**25^	0.181	1.01 × 10^**−**24^
		Area under curve (AUC) (assessor) ^d^	0.769	3.00 × 10^**−**6^	0.853	7.47 × 10^**−**10^	0.769	3.00 × 10^**−**6^
		Area under curve (AUC) (participant)^e^	0.750	5.63 × 10^−10^	0.806	3.02 × 10^**−**14^	0.750	5.63 × 10^**−**10^
Photo-ageing	Dyspigmentation constituent of photo-ageing as measured by the Korean scale	Spearman’s rank correlation (ρ)	0.203	1.63 × 10^**−**11^	0.278	1.44 × 10^**−**20^	0.226	5.54 × 10^**−**14^
		Cohen’s kappa (κ)	0.099	1.09 × 10^**−**10^	0.155	8.34 × 10^**−**22^	0.130	2.28 × 10^**−**12^
		Area under curve (AUC) (assessor) ^d^	0.716	6.65 × 10^**−**7^	0.727	6.89 × 10^**−**4^	0.716	6.65 × 10^**−**7^
		Area under curve (AUC) (participant)^e^	0.683	1.79 × 10^−10^	0.722	1.18 × 10^**−**14^	0.689	4.27 × 10^**−**11^

^a^Lax definition: a phenotype is determined to be present if any of the three assessors identify it

^b^Moderately strict definition: a phenotype is determined to be present if at least two of the three assessors identify it

^c^Strict definition: a phenotype is determined to be present only if all three assessors identify it

^d^Area under curve (assessor): area under the receiver operating characteristic (ROC) curve (AUC) when the grading by the assessor is treated as the putative gold standard

^e^Area under curve (participant): area under the receiver operating characteristic (ROC) curve (AUC) when the grading by the participant is treated as the putative gold standard

Cohen’s kappa (κ) values report the levels of interrater agreement after accounting for agreements due to chance. Cohen’s kappa (κ) is calculated using Version 25 of the IBM Statistical Package for Social Scientists (SPSS/PC) and reported in Tables 2 and S2. The strength of Cohen’s kappa is interpreted as follows: 0.00–0.19: weak, 0.20–0.39: fair, 0.40–0.59: moderate, 0.60–0.79: very strong, and 0.80–1.00: perfect.

**Fig. 1:**
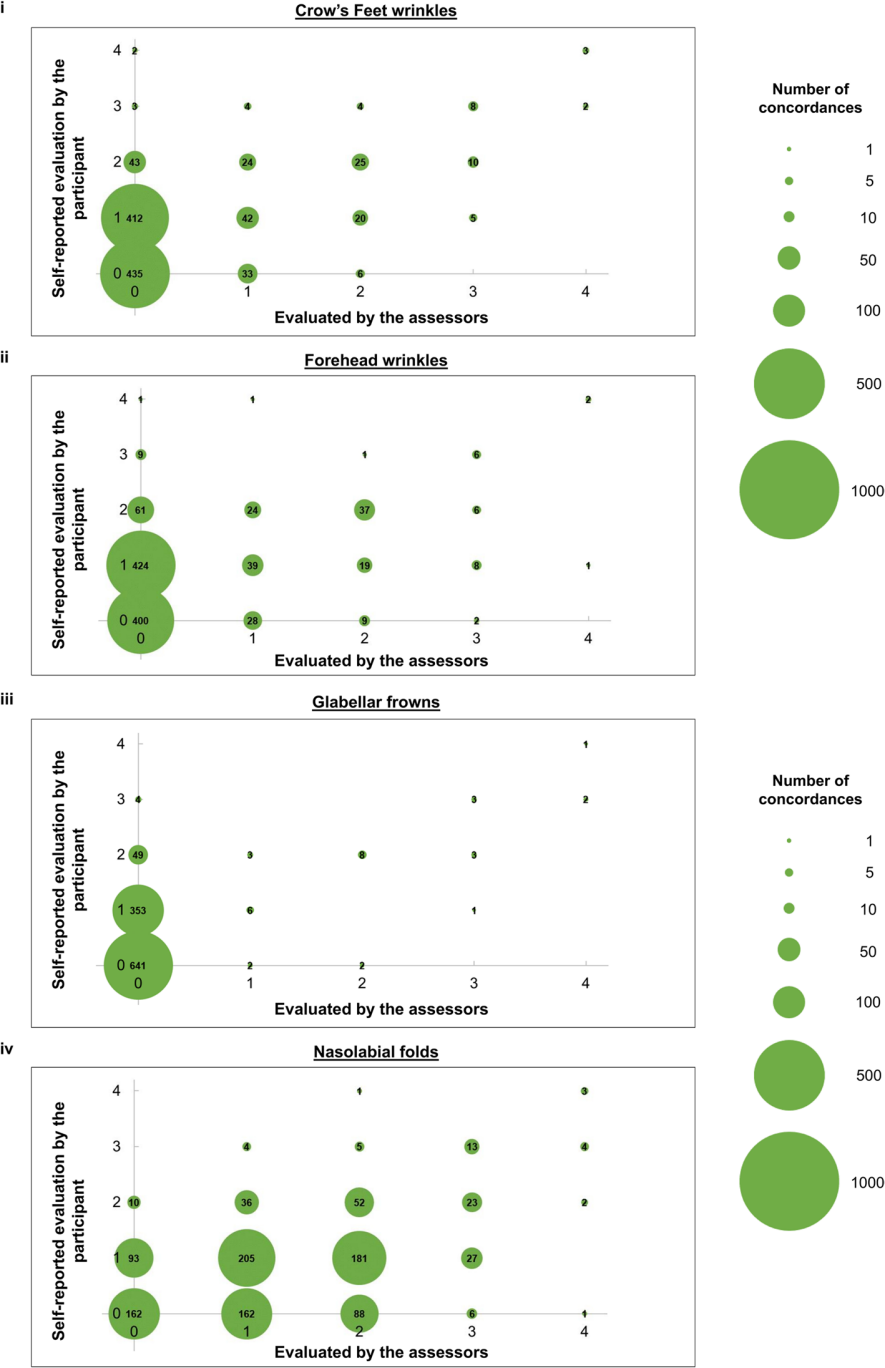
**i** Bubble plots compare the evaluation of Crow’s Feet wrinkles by our three trained assessors and the self-reported evaluation by the participant. For the evaluation performed by our assessors, the phenotype is determined to be present only if all three assessors identify it to be present. Larger circles indicate greater concordance between the two scales. Numbers in the circles are the number of concordances. The sample size of each plot is 1081 participants. **ii** Bubble plots compare the evaluation of forehead wrinkles by our three trained assessors and the self-reported evaluation by the participant. For the evaluation performed by our assessors, the phenotype is determined to be present only if all three assessors identify it to be present. Larger circles indicate greater concordance between the two scales. Numbers in the circles are the number of concordances. The sample size of each plot is 1081 participants. **iii** Bubble plots compare the evaluation of glabellar frowns by our three trained assessors and the self-reported evaluation by the participant. For the evaluation performed by our assessors, the phenotype is determined to be present only if all three assessors identify it to be present. Larger circles indicate greater concordance between the two scales. Numbers in the circles are the number of concordances. The sample size of each plot is 1081 participants. **iv** Bubble plots compare the evaluation of nasolabial folds by our three trained assessors and the self-reported evaluation by the participant. For the evaluation performed by our assessors, the phenotype is determined to be present only if all three assessors identify it to be present. Larger circles indicate greater concordance between the two scales. Numbers in the circles are the number of concordances. The sample size of each plot is 1081 participants

**Fig. 2:**
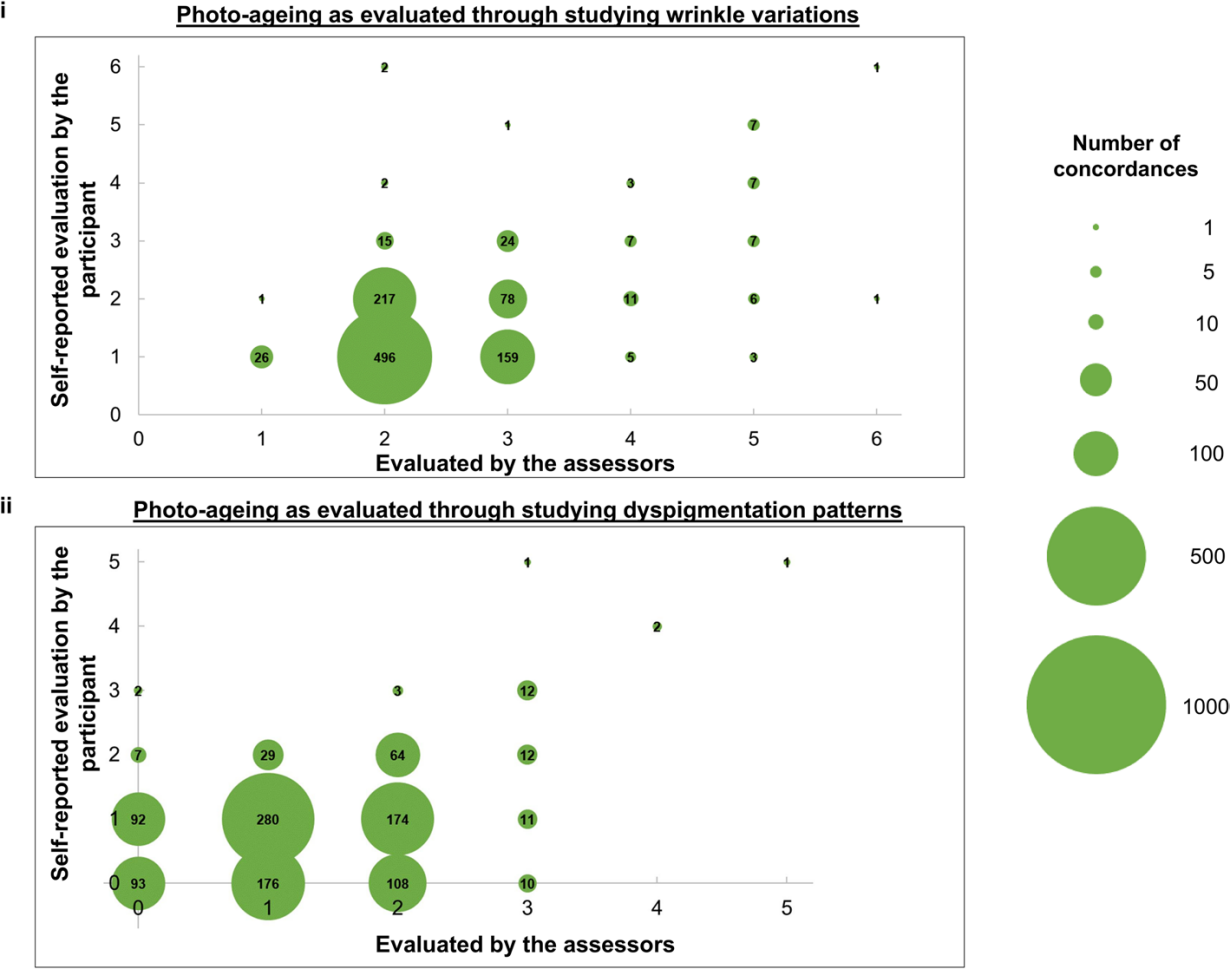
**i** Bubble plots compare photo-ageing as evaluated through studying wrinkle variations. Evaluations conducted by our three trained assessors are compared with the self-reported evaluation by the participant. For the evaluation performed by our assessors, the phenotype is determined to be present only if all three assessors identify it to be present. Larger circles indicate greater concordance between the two scales. Numbers in the circles are the number of concordances. The sample size of each plot is 1081 participants. **ii** Bubble plots compare photo-ageing as evaluated through studying dyspigmentation patterns. Evaluations conducted by our three trained assessors are compared with the self-reported evaluation by the participant. For the evaluation performed by our assessors, the phenotype is determined to be present only if all three assessors identify it to be present. Larger circles indicate greater concordance between the two scales. Numbers in the circles are the number of concordances. The sample size of each plot is 1081 participants

Bubble plots compare the grades for wrinkles (Fig. 1) and photo-ageing (Fig. 2) evaluated by our trained assessors or self-reported by the participant.

**Fig. 3:**
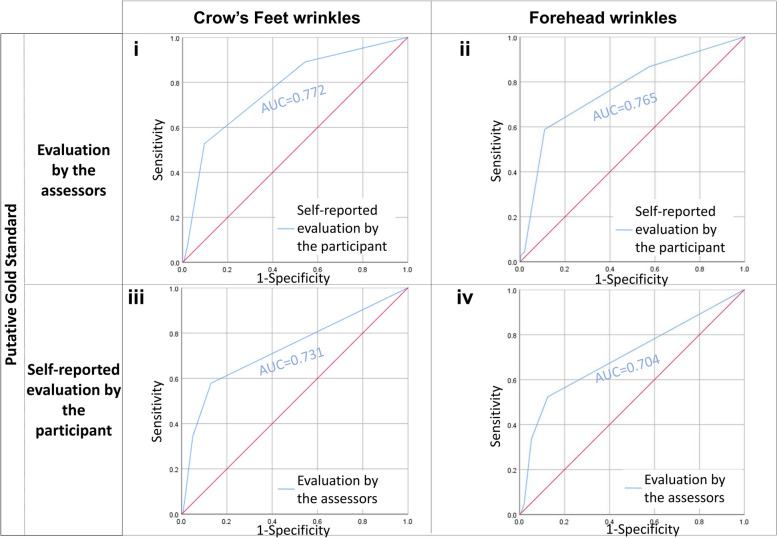
**i** Receiver operator characteristic (ROC) curves treat the evaluation by our three trained assessors as the gold standard and compare it with the self-reported evaluation by the participant. AUC refers to the area under curve values of the corresponding ROC curves. ROC curves describe grading data for Crow’s Feet wrinkles. **ii** Receiver operator characteristic (ROC) curves treat the evaluation by our three trained assessors as the gold standard and compare it with the self-reported evaluation by the participant. AUC refers to the area under curve values of the corresponding ROC curves. ROC curves describe grading data for forehead wrinkles. **iii** Receiver operator characteristic (ROC) curves treat the self-reported evaluation by the participant as the gold standard and compare it with the evaluation by our three trained assessors. AUC refers to the area under curve values of the corresponding ROC curves. ROC curves describe grading data for Crow’s Feet wrinkles. **iv** Receiver Operator Characteristic (ROC) curves treat the self-reported evaluation by the participant as the gold standard and compare it with the evaluation by our three trained assessors. AUC refers to the area under curve values of the corresponding ROC curves. ROC curves describe grading data for forehead wrinkles

**Fig. 4:**
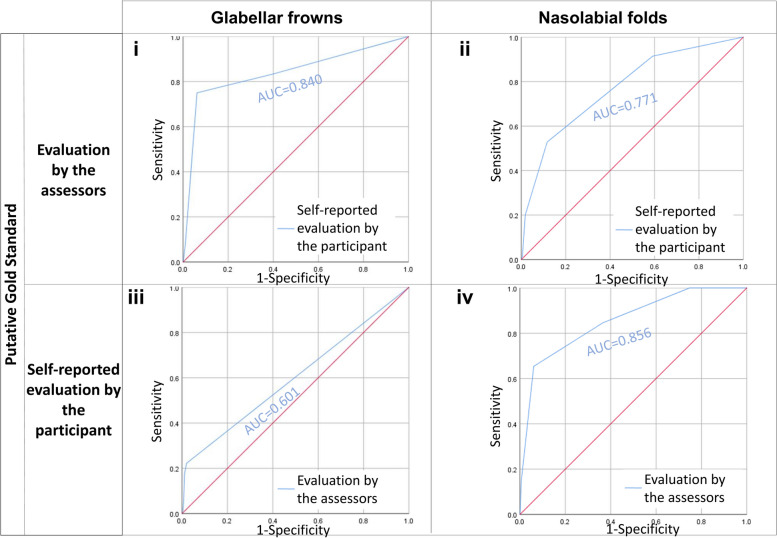
**i** Receiver operator characteristic (ROC) curves treat the evaluation by our three trained assessors as the gold standard and compare it with the self-reported evaluation by the participant. AUC refers to the area under curve values of the corresponding ROC curves. ROC curves describe grading data for glabellar frowns. **ii** Receiver operator characteristic (ROC) curves treat the evaluation by our three trained assessors as the gold standard and compare it with the self-reported evaluation by the participant. AUC refers to the area under curve values of the corresponding ROC curves. ROC curves describe grading data for nasolabial folds. **iii** Receiver operator characteristic (ROC) curves treat the self-reported evaluation by the participant as the gold standard and compare it with the evaluation by our three trained assessors. AUC refers to the area under curve values of the corresponding ROC curves. ROC curves describe grading data for glabellar frowns. **iv** Receiver operator characteristic (ROC) curves treat the self-reported evaluation by the participant as the gold standard and compare it with the evaluation by our three trained assessors. AUC refers to the area under curve values of the corresponding ROC curves. ROC curves describe grading data for nasolabial folds

**Fig. 5:**
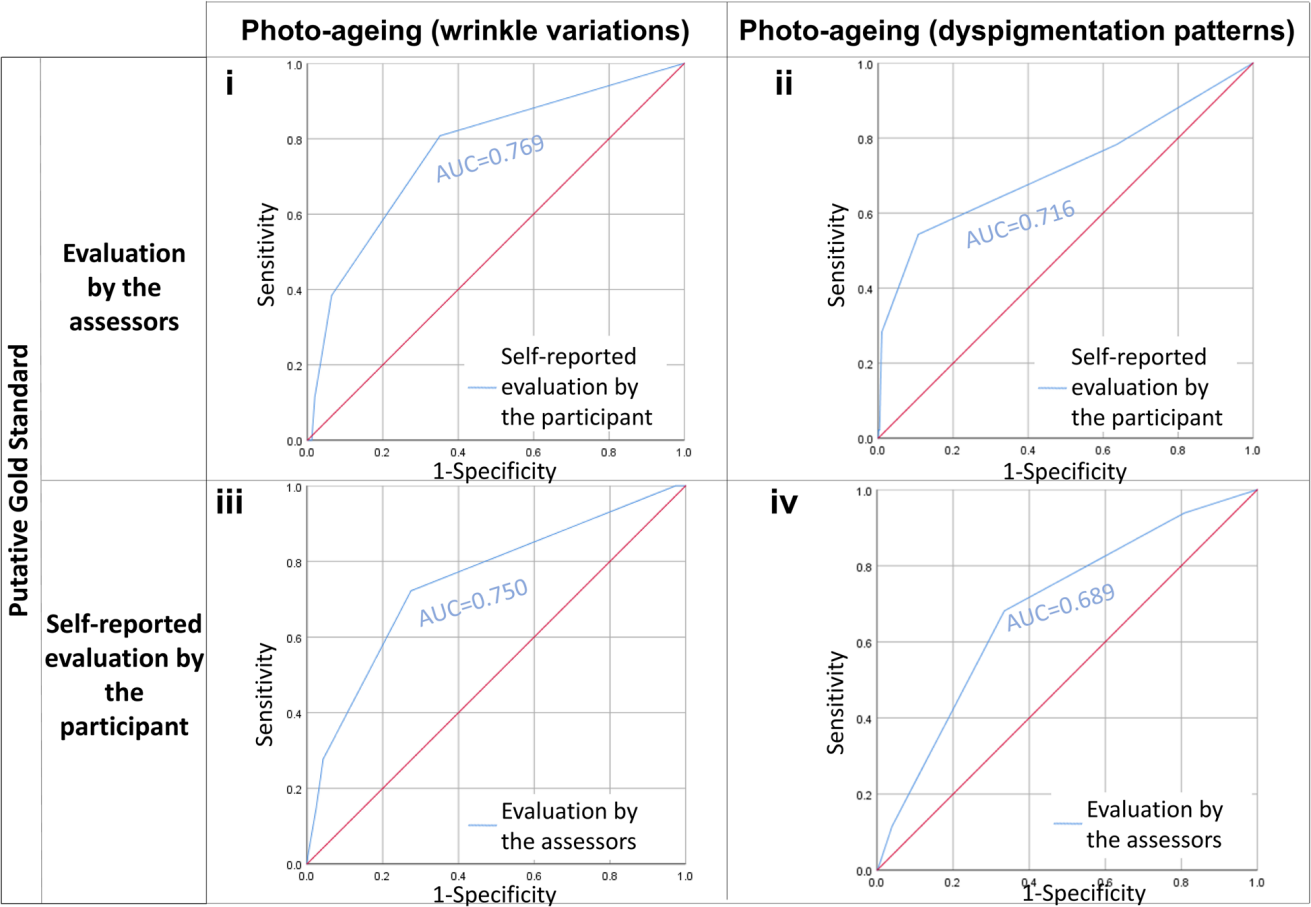
**i** Receiver operator characteristic (ROC) curves treat the evaluation by our three trained assessors as the gold standard and compares it with the self-reported evaluation by the participant. AUC refers to the area under curve values of the corresponding ROC curves. ROC curves describe grading data for photo-ageing as evaluated through studying wrinkle variations. **ii** Receiver operator characteristic (ROC) curves treat the evaluation by our three trained assessors as the gold standard and compare it with the self-reported evaluation by the participant. AUC refers to the area under curve values of the corresponding ROC curves. ROC curves describe grading data for photo-ageing as evaluated through studying dyspigmentation patterns. **iii** Receiver operator characteristic (ROC) curves treat the self-reported evaluation by the participant as the gold standard and compare it with the evaluation by our three trained assessors. AUC refers to the area under curve values of the corresponding ROC curves. ROC curves describe grading data for photo-ageing as evaluated through studying wrinkle variations. **iv** Receiver operator characteristic (ROC) curves treat the self-reported evaluation by the participant as the gold standard and compare it with the evaluation by our three trained assessors. AUC refers to the area under curve values of the corresponding ROC curves. ROC curves describe grading data for photo-ageing as evaluated through studying dyspigmentation patterns

The area under the curve (AUC) of the Receiver Operator Characteristic (ROC) curve is computed using SPSS. AUC curves compare the grades for wrinkles (Figs. 3 and 4) and photo-ageing (Fig. 5) evaluated by our trained assessors or self-reported by the participant. The strength of the AUC is interpreted as follows: 0.51–0.59: very weak, 0.60–0.69: weak, 0.70–0.79: fair, 0.80–0.89: strong, and 0.90–1.00: very strong.

**Fig. 6:**
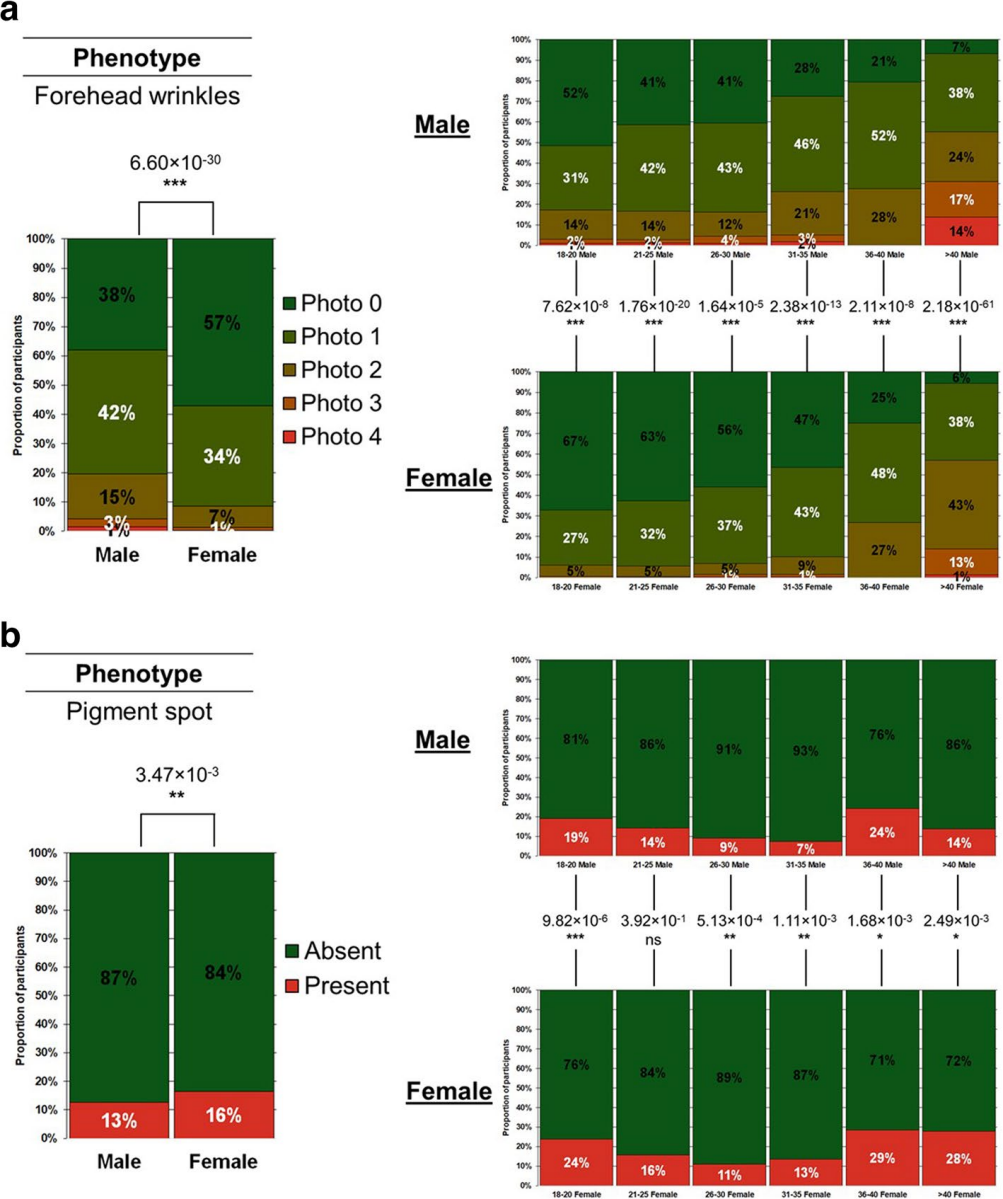
**a** Severity of forehead wrinkles stratified by age and sex. Forehead wrinkles are quantified as a grade on a validated photo-numeric scale. The chi-square test *p* values for all the pair-wise comparisons conducted were corrected for multiple testing through the Bonferroni correction method. The photo-numeric scale used for assessing forehead wrinkles can be found in Supplementary Table S1. **b** Presence or absence of pigment spots stratified by age and sex. Pigment spots are quantified as a binary grade. Photos of pigment spots are obtained from Ferri’s Fast Facts in Dermatology. Participants are deemed to have pigment spots if their skin appear similar to the photos from Ferri's Fast Facts in Dermatology. The chi-square test *p* values for all the pair-wise comparisons conducted were corrected for multiple testing through the Bonferroni correction method. The citation for Ferri’s Fast Facts in Dermatology, in which photos used for assessing pigment spots are available, can be found in Supplementary Table S1

**Fig. 7:**
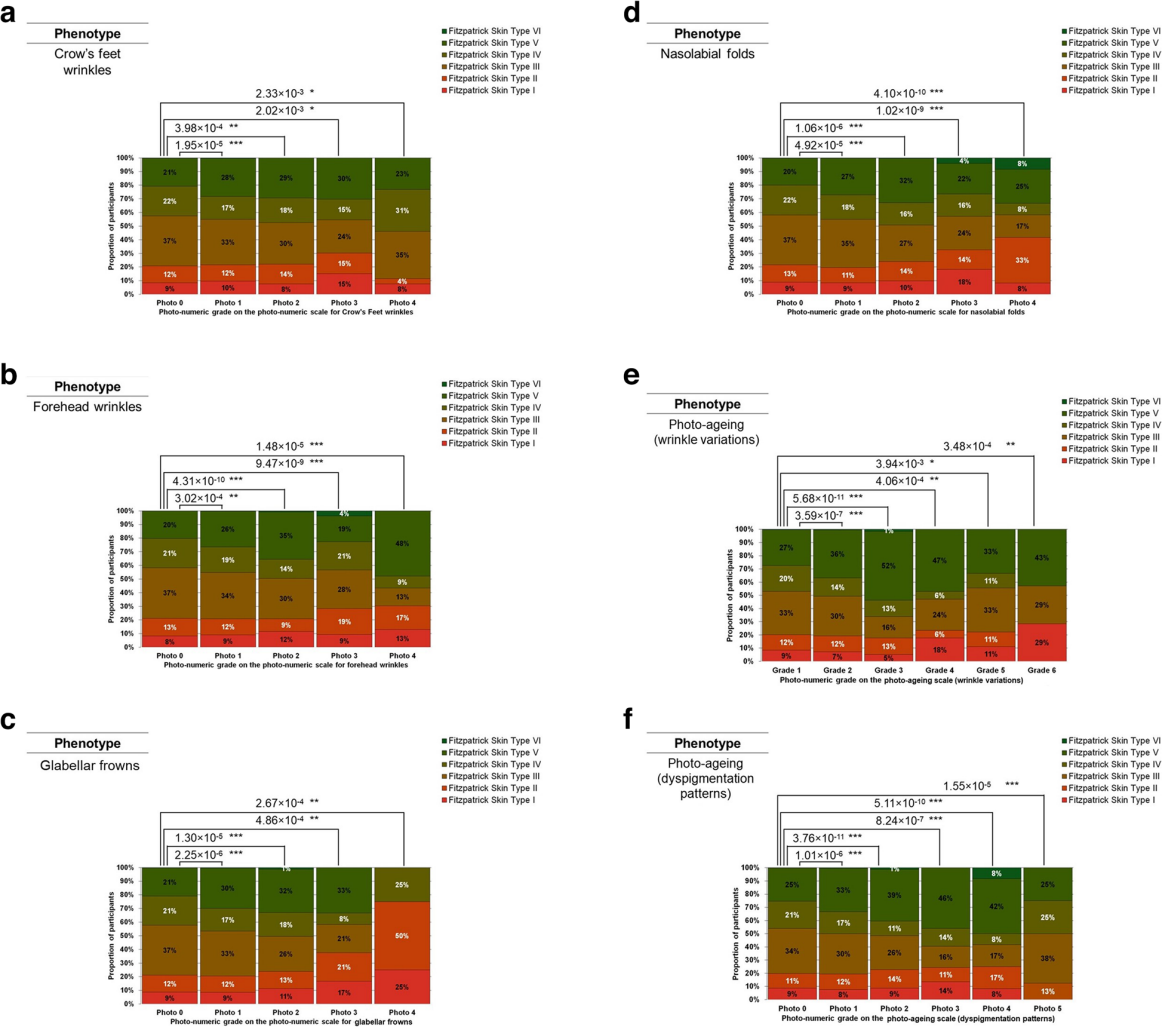
**a** Distribution of Fitzpatrick Skin Types I to VI across participants with different severities of Crow’s Feet wrinkles. Crow’s Feet wrinkles are quantified as a grade on a validated photo-numeric scale. The chi-square test *p* values for all the pair-wise comparisons conducted were corrected for multiple testing through the Bonferroni correction method. The photo-numeric scale used for this phenotypic assessment can be found in Supplementary Table S1. **b** Distribution of Fitzpatrick Skin Types I to VI across participants with different severities of forehead wrinkles. Forehead wrinkles are quantified as a grade on a validated photo-numeric scale. The chi-square test *p* values for all the pair-wise comparisons conducted were corrected for multiple testing through the Bonferroni correction method. The photo-numeric scale used for this phenotypic assessment can be found in Supplementary Table S1. **c** Distribution of Fitzpatrick Skin Types I to VI across participants with different severities of glabellar frowns. Glabellar frowns are quantified as a grade on a validated photo-numeric scale. The chi-square test *p* values for all the pair-wise comparisons conducted were corrected for multiple testing through the Bonferroni correction method. The photo-numeric scale used for this phenotypic assessment can be found in Supplementary Table S1. **d** Distribution of Fitzpatrick Skin Types I to VI across participants with different severities of nasolabial folds. Nasolabial folds are quantified as a grade on a validated photo-numeric scale. The chi-square test *p* values for all the pair-wise comparisons conducted were corrected for multiple testing through the Bonferroni correction method. The photo-numeric scale used for this phenotypic assessment can be found in Supplementary Table S1. **e** Distribution of Fitzpatrick Skin Types I to VI across participants with different severities of the wrinkling constituent of photo-ageing. The wrinkling constituent of photo-ageing is quantified as a grade on a validated photo-numeric scale. The Chi-square test *p*-values for all the pair-wise comparisons conducted were corrected for multiple testing through the Bonferroni correction method. The photo-numeric scale used for this phenotypic assessment can be found in Supplementary Table S1. **f** Distribution of Fitzpatrick Skin Types I to VI across participants with different severities of the dyspigmentation constituent of photo-ageing. The dyspigmentation constituent of photo-ageing is quantified as a grade on a validated photo-numeric scale. The chi-square test *p* values for all the pair-wise comparisons conducted were corrected for multiple testing through the Bonferroni correction method. The photo-numeric scale used for this phenotypic assessment can be found in Supplementary Table S1

**Fig. 8:**
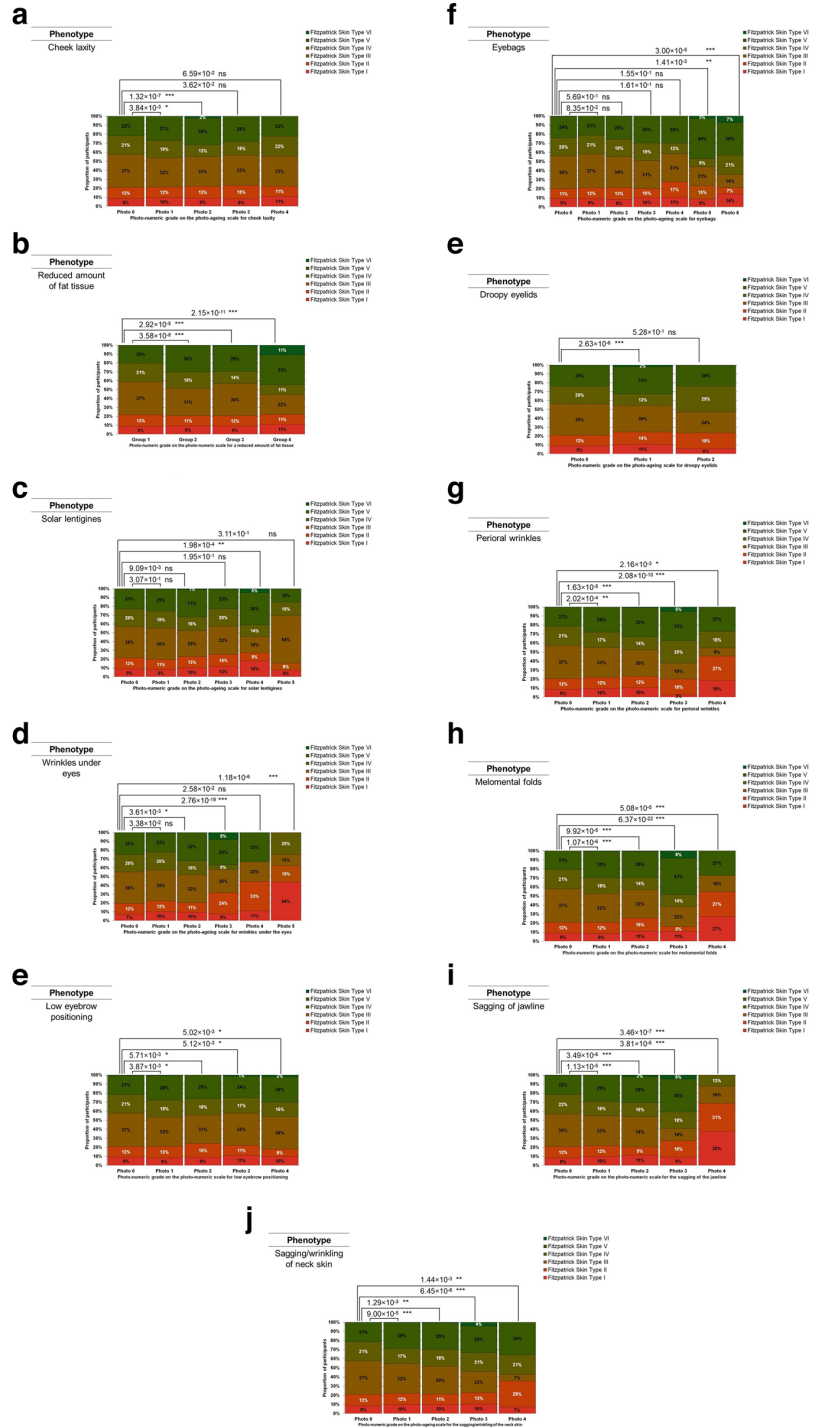
**a** Distribution of Fitzpatrick Skin Types I to VI across participants with different cheek laxities. Cheek laxity is quantified as a grade on a validated photo-numeric scale. The chi-square test *p* values for all the pair-wise comparisons conducted were corrected for multiple testing through the Bonferroni correction method. The photo-numeric scale used for this phenotypic assessment can be found in Supplementary Table S1. **b** Distribution of Fitzpatrick Skin Types I to VI across participants with different amounts of fat tissue. The amount of fat tissue is quantified as a grade on a validated photo-numeric scale. The chi-square test *p* values for all the pair-wise comparisons conducted were corrected for multiple testing through the Bonferroni correction method. The photo-numeric scale used for this phenotypic assessment can be found in Supplementary Table S1. **c** Distribution of Fitzpatrick Skin Types I to VI across participants with different severities of solar lentigines. Solar lentigines are quantified as a grade on a validated photo-numeric scale. The chi-square test *p* values for all the pair-wise comparisons conducted were corrected for multiple testing through the Bonferroni correction method. The photo-numeric scale used for this phenotypic assessment can be found in Supplementary Table S1. **d** Distribution of Fitzpatrick Skin Types I to VI across participants with different severities of wrinkles under the eyes. Wrinkles under the eyes are quantified as a grade on a validated photo-numeric scale. The chi-square test *p* values for all the pair-wise comparisons conducted were corrected for multiple testing through the Bonferroni correction method. The photo-numeric scale used for this phenotypic assessment can be found in Supplementary Table S1. **e** Distribution of Fitzpatrick Skin Types I to VI across participants with different eyebrow positioning heights. Eyebrow positioning heights are quantified as a grade on a validated photo-numeric scale. The chi-square test *p* values for all the pair-wise comparisons conducted were corrected for multiple testing through the Bonferroni correction method. The photo-numeric scale used for this phenotypic assessment can be found in Supplementary Table S1. **f** Distribution of Fitzpatrick Skin Types I to VI across participants with different severities of eyebags. Eyebags are quantified as a grade on a validated photo-numeric scale. The chi-square test *p* values for all the pair-wise comparisons conducted were corrected for multiple testing through the Bonferroni correction method. The photo-numeric scale used for this phenotypic assessment can be found in Supplementary Table S1. **g** Distribution of Fitzpatrick Skin Types I to VI across participants with different severities of droopy eyelids. Droopy eyelids are quantified as a grade on a validated photo-numeric scale. The chi-square test *p* values for all the pair-wise comparisons conducted were corrected for multiple testing through the Bonferroni correction method. The photo-numeric scale used for this phenotypic assessment can be found in Supplementary Table S1. **h** Distribution of Fitzpatrick Skin Types I to VI across participants with different severities of perioral wrinkles. Perioral wrinkles are quantified as a grade on a validated photo-numeric scale. The chi-square test *p* values for all the pair-wise comparisons conducted were corrected for multiple testing through the Bonferroni correction method. The photo-numeric scale used for this phenotypic assessment can be found in Supplementary Table S1. **i** Distribution of Fitzpatrick Skin Types I to VI across participants with different severities of Melomental folds. Melomental folds are quantified as a grade on a validated photo-numeric scale. The chi-square test *p* values for all the pair-wise comparisons conducted were corrected for multiple testing through the Bonferroni correction method. The photo-numeric scale used for this phenotypic assessment can be found in Supplementary Table S1. **j** Distribution of Fitzpatrick Skin Types I to VI across participants with different severities of jawline sagging. Jawline sagging is quantified as a grade on a validated photo-numeric scale. The chi-square test* p* values for all the pair-wise comparisons conducted were corrected for multiple testing through the Bonferroni correction method. The photo-numeric scale used for this phenotypic assessment can be found in Supplementary Table S1. **k** Distribution of Fitzpatrick Skin Types I to VI across participants with different severities of sagging and/or wrinkling of the neck skin. Sagging and/or wrinkling of the neck skin is quantified as a grade on a validated photo-numeric scale. The chi-square test *p* values for all the pair-wise comparisons conducted were corrected for multiple testing through the Bonferroni correction method. The photo-numeric scale used for this phenotypic assessment can be found in Supplementary Table S1

Chi-square tests are performed to evaluate the proportion of participants with skin ageing phenotypes at different severity levels (Figs. 6, 7, and 8). Chi-square trend tests are performed to evaluate whether the changes in these proportions follow a significant trend with chronological age in both sexes.

## Results

### Participant demographics

We studied a group of 1081 participants which can provide a representative overview of the epidemiology of skin ageing in the Singapore ethnic Chinese young adult population. This group has been studied in detail in our previous works (Ng JY, & Chew FT. Comparisons between Caucasian validated photo-numeric scales and Chinese validated photo-numeric scales for assessing facial wrinkles: Crow’s Feet wrinkles, forehead wrinkles, glabellar frowns, and nasolabial folds. Insights from the Singapore/Malaysia Cross-sectional Genetics Epidemiology Study (SMCGES) cohort. Manuscript submitted for publication in Scientific Reports) (Ng JY, & Chew FT. Comparisons between Caucasian validated photo-numeric scales and Korean validated photo-numeric scales for photo-ageing. Insights from the Singapore/Malaysia Cross-sectional Genetics Epidemiology Study (SMCGES) cohort. Manuscript submitted for publication in Skin Research and Technology). Briefly, this group consists of more females (*n* = 661, 61.15%) than males. The average age of the participants is 26.15 ± 8.11 years old. Our participants are 166.03 ± 8.60 cm in height, 60.96 ± 12.60 kg in weight, and have a BMI of 22.02 ± 3.99 kg/m^2^. Most participants have a total monthly family income per capita of ≥ 6000 Singapore dollars (SGD) (*n* = 446, 41.26%) and most participants stay in HDB public housing (*n* = 664, 61.42%) (Table 1).

### Validated photo-numeric scales for wrinkling and photo-ageing

We have previously shown that Caucasian scales for four types of facial wrinkles (Crow’s Feet wrinkles, forehead wrinkles, glabellar frowns, and nasolabial folds) (Table S1) are suitable photo-numeric scales to evaluate facial wrinkles on Chinese skin (Ng JY, & Chew FT. Comparisons between Caucasian validated photo-numeric scales and Chinese validated photo-numeric scales for assessing facial wrinkles: Crow’s Feet wrinkles, forehead wrinkles, glabellar frowns, and nasolabial folds. Insights from the Singapore/Malaysia Cross-sectional Genetics Epidemiology Study (SMCGES) cohort. Manuscript submitted for publication in Scientific Reports). We have also previously identified that the Larnier scale and a Korean dyspigmentation scale (Table S1) are the most suitable scales currently available to identify photo-ageing on ethnic Chinese skin (Ng JY, & Chew FT. Comparisons between Caucasian validated photo-numeric scales and Korean validated photo-numeric scales for photo-ageing. Insights from the Singapore/Malaysia Cross-sectional Genetics Epidemiology Study (SMCGES) cohort. Manuscript submitted for publication in Skin Research and Technology). Here, we will study these phenotypes further and assess how these scales perform when used by a trained assessor and an untrained participant.

### Adjusting for potential variations in evaluation standards

We designed a three-tiered stringency-based classification model for the identification of wrinkles and photo-ageing (Table 2). This is done to introduce flexibility and account for variation between assessors. At the laxest level of stringency, a skin ageing phenotype (e.g., Crow’s Feet wrinkles) is determined to be present if any of the three assessors identify it. At the other end of the spectrum, the strictest level of stringency requires all three assessors to identify the phenotype in order to treat the phenotype as present. We also examined the middle ground (i.e., a moderately strict level of stringency) in which a skin ageing phenotype is determined to be present when at least two of the three assessors identify it.

### Wrinkles

Our main objective is to use the validated photo-numeric scale identified in “Validated photo-numeric scales for wrinkling and photo-ageing” section and compare whether there are differences when wrinkles and photo-ageing are identified by trained assessors and by untrained participants. Here, we report the results for four wrinkle types: Crow’s Feet wrinkles, forehead wrinkles, glabellar frowns, and nasolabial folds. The concordance between self-reported Crow’s Feet wrinkles and Crow’s Feet wrinkles identified by our trained assessors is fair (Spearman correlation = 0.281–0.346, *p* value ≤ 4.64 × 10^−21^) (Table 2, Fig. 1i). After adjusting for agreements due to chance, we still find a significant level of agreement between the participant and our assessors (Cohen’s kappa = 0.101–0.180, *p* value ≤ 1.00 × 10^−11^) (Table 2). When the evaluation by our trained assessors is treated as the gold standard, the positive identification of Crow’s Feet wrinkles by our participants ranges from weak to fair (AUC = 0.671–0.798, *p* value ≤ 1.79 × 10^−11^). Taking the evaluation by the participant as the gold standard, our assessors identified Crow’s Feet wrinkles similarly well (AUC = 0.659–0.731, *p* value ≤ 1.16 × 10^−7^) (Table 2, Fig. 3).

The concordance (Spearman correlation = 0.286–0.348, *p* value ≤ 4.90 × 10^−25^) (Table 2, Fig. 1ii) observed between the identification of forehead wrinkles by our participants and by our assessors is also fair and is very similar to the results for Crow’s Feet wrinkles reported above. Here, the level of agreement between the participant and our assessors is weak-to-fair (Cohen’s kappa = 0.105–0.233, *p* value ≤ 2.75 × 10^−12^) (Table 2). When taking the evaluation by our assessors as the gold standard, the capability of our participants to identify forehead wrinkles is on average, fairly strong (AUC = 0.671–0.828, *p* value ≤ 3.41 × 10^−11^). Using the self-reported forehead wrinkles as the gold standard, our assessors identified forehead wrinkles similarly well (AUC = 0.686–0.704, *p* value ≤ 7.01 × 10^−12^) (Table 2, Fig. 3).

A fair level of concordance is observed between glabellar frowns identified by our participants and glabellar frowns identified by our assessors (Spearman correlation = 0.249–0.289, *p* value ≤ 9.85 × 10^−17^) (Table 2, Fig. 1iii). The level of agreement for glabellar frowns is weaker (Cohen’s kappa = 0.065–0.170, *p* value ≤ 6.27 × 10^−13^) than the level of agreement for Crow’s Feet wrinkles and forehead wrinkles (Table 2). However, different grades of glabellar frowns can be very strongly distinguished apart from one another by our participants (AUC = 0.795 − 0.911, *p* value ≤ 8.70 × 10^−5^) and weakly by our assessors (AUC = 0.601 − 0.615, *p* value ≤ 6.85 × 10^−3^) (Table 2, Fig. 4).

Lastly, the concordance between self-reported and assessor-evaluated nasolabial folds is the highest (Spearman correlation = 0.339–0.366, *p* value ≤ 1.55 × 10^−30^) (Table 2, Fig. 1iv). The level of agreement for nasolabial folds is similar to that for glabellar frowns (Cohen’s Kappa = 0.053–0.190, *p* value ≤ 9.65 × 10^−4^. Both the participant (AUC = 0.839 − 0.877, *p*-value ≤ 3.49 × 10^−9^) and the assessors (AUC = 0.771–0.847, *p* value ≤ 3.17 × 10^−14^) can strongly distinguish apart different severity grades of nasolabial folds when either of them is taken as the gold standard (Table 2, Fig. 4).

Overall, we interpret our results to indicate that self-reported wrinkles (Crow’s Feet wrinkles, forehead wrinkles, glabellar frowns, and nasolabial folds) evaluated through Caucasian scales satisfactorily approximate the wrinkle grades evaluated by our assessors with a fair level of concordance.

### Photo-ageing

We found that self-reported photo-ageing is concordant with photo-ageing identified by our trained assessors (Table 2, Fig. 2). We have earlier shown that photo-ageing is best evaluated separately through the wrinkling constituent and the dyspigmentation constituent (Ng JY, & Chew FT. Comparisons between Caucasian validated photo-numeric scales and Korean validated photo-numeric scales for photo-ageing. Insights from the Singapore/Malaysia Cross-sectional Genetics Epidemiology Study (SMCGES) cohort. Manuscript submitted for publication in Skin Research and Technology).

We find that the level of concordance for the wrinkling constituent of photo-ageing between self-reported data and data identified by our trained assessors (Spearman correlation = 0.246–0.383, *p* value ≤ 2.24 × 10^−16^) is fair and significant. Our participants and assessors also significantly agree with one another (Cohen’s kappa = 0.041–0.181, *p* value ≤ 9.80 × 10^−5^). When we take the evaluation by our assessors as the gold standard, our participants are fair-to-strongly capable of discerning the different levels of photo-ageing and identifying the most appropriate level of severity which corresponds to their skin (AUC = 0.769–0.853, *p* value ≤ 3.00 × 10^−6^) (Table 2, Fig. 5). When we take the evaluation by the participants as the gold standard instead, we find similar results (AUC = 0.750–0.806, *p* value ≤ 5.63 × 10^−10^) (Table 2, Fig. 5). This evidence suggests that the photo-ageing scale used to assess the wrinkling constituent of photo-ageing is sufficiently flexible to be used either by a trained assessor or an untrained participant.

Meanwhile, our participants and our assessors are also concordant with one another in grading the dyspigmentation constituent of photo-ageing (Spearman correlation = 0.203–0.278, *p* value ≤ 1.63 × 10^−11^). The level of concordance between the participants and assessors for the dyspigmentation constituent of photo-ageing is both fair and significant. As above, our participants and assessors also significantly agree with one another (Cohen’s kappa = 0.099–0.155, *p* value ≤ 1.09 × 10^−10^). Taking the evaluation by our assessors as the gold standard, participants are fairly capable of distinguishing apart different grades of dyspigmentation and locating the severity level which best resembles their skin (AUC = 0.716–0.727, *p* value ≤ 6.89 × 10^−4^) (Table 2, Fig. 5). Likewise, when using the evaluation by our participants as the gold standard, we find that our assessors often identify the same severity grade as the participants (AUC = 0.683–0.722, *p* value ≤ 1.79 × 10^−10^) (Table 2, Fig. 5).

Overall, our results lead us to interpret that self-reported photo-ageing levels on the Larnier scale and a Korean dyspigmentation scale give satisfactory approximations to the photo-ageing grades evaluated by our assessors as both datasets have a fair level of concordance.

## Discussion

### Participant demographics

Singapore and Malaysia are racially diverse and composed of people of different ethnicities. Tapping on this diversity, we were able to recruit participants from different ethnicities. Nonetheless, there is an unequal proportion and sampling probability among some races, with only ethnic Chinese remaining as the major racial group with a large enough sample size. In this study, we focus on the ethnic Chinese; however, future analyses and descriptions of skin ageing in other races (e.g., Malays and Indians) will be conducted when the number of participants of the other races grows sufficiently large through progressive, annual recruitment drives to expand the Singapore/Malaysia Cross-section Genetics Epidemiology Study (SMCGES) cohort.

### Comparing self-reported phenotypes with the evaluations from our trained assessors

Skin ageing is a continuous process. One of the ways to quantify skin ageing uses a series of validated photo-numeric scales. Each photo-numeric scale consists of a set of photographs and successive photographs show increased intensities of ageing either on a specific area of the skin or on the overall appearance of the skin. Our previous work on wrinkles (Ng JY, & Chew FT. Comparisons between Caucasian validated photo-numeric scales and Chinese validated photo-numeric scales for assessing facial wrinkles: Crow’s Feet wrinkles, forehead wrinkles, glabellar frowns, and nasolabial folds. Insights from the Singapore/Malaysia Cross-sectional Genetics Epidemiology Study (SMCGES) cohort. Manuscript submitted for publication in Scientific Reports) and photo-ageing (Ng JY, & Chew FT. Comparisons between Caucasian validated photo-numeric scales and Korean validated photo-numeric scales for photo-ageing. Insights from the Singapore/Malaysia Cross-sectional Genetics Epidemiology Study (SMCGES) cohort. Manuscript submitted for publication in Skin Research and Technology) have shown that the same skin is consistently evaluated to have the same severity grade on the same photo-numeric scale regardless of who is making the assessment. This led us to wonder how the signs of skin ageing which are identified by the participant compare with those which are identified by our trained assessors. As participants are not specially trained to identify the signs of ageing on their skin, it is possible to encounter considerable variations in the assessments made by different participants. Well-designed photo-numeric scales would be better equipped to address such variations.

Our main objective here is to examine how photo-numeric scales perform using self-reported data as compared to using data evaluated by our trained assessors. By making these comparisons, we aim to understand whether self-reported signs of skin ageing provide a satisfactory approximation to the signs of skin ageing identified by our assessors. To make the comparisons fair across the entire spectrum of participants, we decided that it is not as meaningful to compare the self-reported data with the mean gradings given by our trained assessors. Instead, we adapted the scores from our trained assessors into a three-tiered stringency-based classification model (Table 2). The three tiers are lax, moderately strict, and strict. Each tier had been described earlier in the “Adjusting for potential variations in evaluation standards” section. Briefly, three datasets are derived from the assessors and each dataset corresponds to one of the three tiers. The three datasets use different thresholds to determine whether a phenotype is present or absent. In the dataset with the laxest level of stringency, a skin ageing phenotype (e.g., Crow’s Feet wrinkles) is determined to be present if any of the three assessors identify it. The moderately-strict dataset is considerably more stringent in which a skin ageing phenotype is determined to be present if at least two of the three assessors identify it. Lastly, the strict dataset gives the highest level of stringency because here, a skin ageing phenotype is determined to be present only if all three assessors identify it (Table 2).

We found that taking a moderately strict approach to identifying signs of photo-ageing delivers the best results: Spearman correlation between self-reported data and the evaluation by our trained assessors is fairly strong (Spearman correlation ρ = 0.383, *p* = 4.86 × 10^−39^) for the wrinkling constituent of photo-ageing and is also fairly strong (Spearman correlation ρ = 0.278, *p* = 1.44 × 10^−20^) for the dyspigmentation constituent of photo-ageing (Table 2). Taking a moderately strict approach is also more effective in identifying forehead wrinkles (Spearman correlation ρ = 0.348, *p* = 4.34 × 10^−32^) and glabellar frowns (Spearman correlation ρ = 0.289, *p* = 2.92 × 10^−22^). Meanwhile, Crow’s Feet wrinkles (Spearman correlation ρ = 0.346, *p* = 1.04 × 10^−31^) and nasolabial folds (Spearman correlation ρ = 0.366, *p* = 1.42 × 10^−35^) are more concordant with self-reported data when they are graded using a strict approach (Table 2). We extended our stringency-based classification model to other signs of skin ageing (e.g., wrinkling in other areas, sagging, dyspigmentation) and observed similar results (Table S2). We found that the moderately strict and strict levels of stringency in identifying a phenotype yield the highest concordance with self-reported signs of skin ageing.

Overall, we interpret our findings to be indicative that participants often recognise and report that they possess the same skin ageing phenotype identified by our assessors. We found that the behaviour in which participants report the presence of a given sign of ageing on their skin can be best captured by the middle tier of our three-tiered stringency-based classification model. Putting it another way, most participants are self-aware of ageing signs on their skin early on, especially so when such signs are noticeable by more than one independent assessor.

While the specific behaviour of the data varies from phenotype to phenotype, the bottom line remains the same: signs of skin ageing identified by the participant are concordant with signs of skin ageing identified by our assessors using the same photo-numeric scale. These concordances are significant and persist even after we use methods to account for any differences in evaluation standards. Self-reported signs of skin ageing thus provide a satisfactory approximation to the signs of skin ageing identified by our assessors.

### Interpreting the statistical indices

We attempted to evaluate six different facets of skin ageing by comparing self-reported data with assessor-evaluated data for six different skin ageing phenotypes. Our primary goal is to characterise how the ascertainment of skin ageing phenotypes differs among different people.

The six skin ageing phenotypes were evaluated using photo-numeric scales. Each of the four wrinkle phenotypes (Crow’s Feet wrinkles, forehead wrinkles, glabellar frowns, nasolabial folds) and two photo-ageing phenotypes (the wrinkling constituent of photo-ageing, and the dyspigmentation constituent of photo-ageing) were evaluated on a dedicated scale.

As skin ageing is a multi-faceted phenomenon, a thorough understanding of skin ageing requires us to take a multi-pronged approach. Here, we will discuss three statistical tools used in our current assessment to better characterise and understand how the ascertainment of skin ageing phenotypes differs among different people.

#### Spearman correlation coefficients

First, we investigated the correlation between Crow’s Feet wrinkles ascertained by our assessors and self-reported by our participants (Fig. 1i). We have established in previous work that photo-numeric scales are the superior method for ascertaining skin ageing phenotypes [18]. By their nature, photo-numeric scales comprise a sequence of photographs showing a stepwise increase in the severity of skin ageing phenotypes (e.g., Crow’s Feet wrinkles) between successive photographs. Spearman correlation measures the association between two categorical ordinal variables. Since the ascertainment of Crow’s Feet wrinkles is done by both the assessor and the participant on the same photo-numeric scale, both evaluations are categorical and ordinal in nature. Spearman correlation is an appropriate tool to numerically describe this association. The numerical value of Spearman’s ρ ranges from 0 to 1, in which 0 implies no correlation and 1 implies complete correlation. While it is understood that values closer to 0 show a weak correlation and values closer to 1 show a strong correlation, the naming practices for the strength of Spearman correlation coefficients vary from field to field [1, 5]. We applied the qualitative interpretations used in healthcare and related fields to interpret Spearman correlation values on skin ageing phenotypes. The strengths are interpreted as follows: 0.00: no correlation, 0.01–0.20: weak, 0.21–0.50: fair, 0.51–0.70: moderate, 0.71–0.90: very strong, 0.91–1.00: perfect. Our results indicate that the strength of Spearman correlations between self-reported data and assessor-evaluated data is fair for the six different facets of skin ageing studied in this paper.

Next, we take a closer look at the bubble plots illustrating the concordance between phenotypes ascertained by the assessor and the participant for Crow’s Feet wrinkles (Fig. 1i), forehead wrinkles (Fig. 1ii), and glabellar frowns (Fig. 1iii). Bubbles that lie on the (0,0), (1,1), (2,2), (3,3), and (4,4) coordinates indicate agreements in the severity of the phenotype. For instance, a value of 435 at the (0,0) coordinate for Crow’s Feet wrinkles indicates that 435 participants self-reported that they do not have Crow’s Feet wrinkles. Crow’s Feet wrinkles were also ascertained as absent by our trained assessors in the same 435 participants (Fig. 1i).

Bubbles at all the other coordinates indicate a difference in judgement between the severity of the phenotype. The larger the difference between the two numbers (e.g., (0,2) compared to (0,1)), the greater the disparity in the assessments. We report 412 participants at the (0,1) coordinate. This means that 412 participants self-reported that they have Crow’s Feet wrinkles, but these Crow’s Feet wrinkles are just barely present. Our trained assessors ascertained that Crow’s Feet wrinkles were absent in the same 412 participants.

Crow’s Feet wrinkles are found near the eyes. As explained in our previous work [18], participants have a very keen awareness of the state of the skin around their eyes. This could explain why a large number of participants picked up on very fine and barely visible Crow’s Feet wrinkles when the assessors did not. This illustrates one of the key strengths of self-reported data: it enables phenotypes to be swiftly detected as soon as they emerge. The main trade-off is that these 412 evaluations lower the Spearman correlation coefficient.

In contrast to 412 participants at the (0,1) coordinate, there are only 43 participants at the (0,2) coordinate, 3 participants at the (0,3) coordinate and 2 participants at the (0,4) coordinate. This indicates that once a wrinkle has become somewhat visible, it will almost certainly be detected by our assessors and graded accordingly. In other words, our assessors reliably detect the majority of wrinkles, with the only exception being very fine, barely present wrinkles on the verge of emergence.

Upon looking at the bubble plots illustrating the concordance between phenotypes ascertained by the assessor and the participant for nasolabial folds (Fig. 1iv), the wrinkling constituent of photo-ageing (Fig. 2i) and the dyspigmentation constituent of photo-ageing (Fig. 2ii), we see that the assessors are stricter than the participants. Using the dyspigmentation constituent of photo-ageing as an example, there are 176 participants at the (1,0) coordinate compared to just 92 participants at the (0,1) coordinate. This means that our trained assessors ascertained that there is some dyspigmentation in 176 participants. The same 176 participants self-reported that they do not have dyspigmentation (Fig. 2ii). One possible explanation of our results is that the photo-numeric scale for the dyspigmentation constituent of photo-ageing puts equal emphasis on both the number and the size of pigment spots. Participants may have believed that they had pigment spots only if such spots exceeded a certain quantity or size. As a result, the number of participants ascertained to have dyspigmentation patterns is higher than the self-reported numbers. One method to potentially resolve this issue is to separate the evaluation of dyspigmentation patterns into two parts: quantity and size.

In summary, a close evaluation of the data shows that the main contributing factor that lowered the Spearman correlation coefficient for wrinkling phenotypes is a fine line between whether a wrinkle is absent or just barely present and on the verge of emergence. The main contributing factor that lowered the Spearman correlation coefficient for the dyspigmentation constituent of photo-ageing is a decision call on whether many small pigment spots and a few large pigment spots should be classified as the same grade.

Beyond Spearman correlation, we also looked at other statistical indices so that our data can be described in a more thorough manner.

#### Cohen’s kappa coefficients

The second statistical tool we used to characterise how the ascertainment of skin ageing phenotypes differs among different people is Cohen’s kappa (κ). Cohen’s kappa is a more robust measure of inter-rater agreement. It is superior to calculating the percentage of agreement between two raters because Cohen’s kappa accounts for agreements due to chance [14]. The numerical value of Cohen’s kappa ranges from 0 to 1, in which 0 implies no agreement and 1 implies complete agreement.

The Cohen’s kappa for the six different facets of skin ageing studied in this paper lies between weak and fair. The behaviour of this data can be understood using Crow’s Feet wrinkles as an example. As the bulk of our participants are in their mid-twenties (Table 1) where wrinkles are just beginning to emerge, it is challenging to discern between very fine, barely visible wrinkles, and no wrinkles at all. Upon a closer look at the bubble plot for Crow’s Feet wrinkles (Fig. 1i), most of our participants self-reported either no Crow’s Feet wrinkles, or very fine, barely visible Crow’s Feet wrinkles. These two groups have roughly equal proportions. In contrast, our trained assessors are more cautious and evaluated that most of our participants have no Crow’s Feet wrinkles. By superimposing these two distribution charts, we get perfect agreement on the absence of Crow’s Feet wrinkles for approximately half of our participants, and a small discrepancy (i.e., a fine line between no wrinkles and very fine, barely visible wrinkles) for approximately the other half of our participants.

The value of Cohen’s kappa decreases as long as there is a discrepancy, be it a small discrepancy or a large discrepancy. From the perspective of this metric, agreements and disagreements happen in a roughly 1-to-1 ratio. As a result, the value of Cohen’s kappa is small. At first glance, it may be tempting to interpret that there is a weak agreement between skin ageing phenotypes ascertained by the participant and the assessors. However, a closer look reveals that the scale of the disagreements is mostly minuscule. In summary, a close evaluation of the data shows that the main contributing factor to a low Cohen’s Kappa is still just a fine line between whether a wrinkle is absent or just barely present.

#### Area under the curve (AUC) of the receiver operator characteristic (ROC) curve

To be thorough in our analysis, we also looked at the AUC of the ROC curve. The numerical value of AUC ranges from 0.51 to 1.00. The closer the value is to 1.00, the higher the sensitivity and specificity.

Our results indicate that the AUC curves for self-reported data and assessor-evaluated data are similar no matter whether the assessors or the participant is treated as the gold standard.

We interpret that sensitivity and specificity for the ascertainment of Crow’s Feet wrinkles (AUC = 0.659–0.731, *p* value ≤ 1.16 × 10 − ^7^), forehead wrinkles (AUC = 0.686–0.704, *p* value ≤ 7.01 × 10^−12^), glabellar frowns (AUC = 0.601–0.615, *p* value ≤ 6.85 × 10^−3^), nasolabial folds (AUC = 0.771–0.847, *p* value ≤ 3.17 × 10^−14^), the wrinkling constituent of photo-ageing (AUC = 0.750–0.806, *p* value ≤ 5.63 × 10^−10^), and the dyspigmentation constituent of photo-ageing (AUC = 0.683–0.722, *p* value ≤ 1.79 × 10^−10^) can be summarised qualitatively as fair, fairly strong, weak, strong, strong, and fair respectively.

The AUC measures the accuracy of diagnostic tests. In our earlier analyses of the other statistical indices, we have established that our assessors reliably detect the majority of wrinkles, except very fine, barely present wrinkles on the verge of emergence. This is represented graphically by the ROC in which the sensitivity and specificity of detecting a wrinkle increases with the severity of the wrinkle. The size and shape of the AUC changes in tandem with the ROC.

Our data shows that except for glabellar frowns, treating self-reported data as the gold standard for different facets of skin ageing is a suitable approach because this approach has high sensitivity and high specificity.

In summary, we ascertained six different facets of skin ageing by comparing self-reported data with assessor-evaluated data. We derived three statistical indices to summarise the behaviour of the data. Each of the three indices provides a different perspective on the behaviour of data. While some skin ageing phenotypes do not perform that well on one index (e.g., Spearman correlation values), they perform well on another index (e.g., AUC). Hence, it is necessary to look at the data from multiple perspectives in order to give a balanced assessment of the suitability of using self-reported skin ageing data.

### Cross-validating the self-reported dataset with existing literature

To further explore the suitability of using self-reported skin ageing data, we also cross-validated age- and sex-dependent trends from our self-reported dataset with existing literature. To enable a common basis for comparison, we looked specifically at the literature on Asian skin. Here, we will demonstrate our comparisons using two morphologically distinct skin ageing phenotypes: forehead wrinkles (Fig. 6a) and pigment spots (Fig. 6b). We found that the trends from our self-reported dataset and the trends from the literature are the same.

It has been reported that Japanese men below the age of 65 develop more severe forehead wrinkles than age-matched Japanese women [22]. We report that the ascertainment of forehead wrinkles through self-reported means also returns the same trend in our SMCGES cohort. Overall, 62% of Chinese males in our current assessment report some degree of forehead wrinkles while just 43% of Chinese females report the same (*p* value = 6.60 × 10^−30^) (Fig. 6a). When we stratify the data by age, we observe that males in each age group have significantly more severe forehead wrinkles than age-matched females. Males aged 18 − 20 (*p* value = 7.62 × 10^−8^), aged 21 − 25 (*p* value = 1.76 × 10^−20^), aged 26 − 30 (*p* value = 1.64 × 10^−5^), aged 31 − 35 (*p* value = 2.38 × 10^−13^), aged 36 − 40 (*p* value = 2.11 × 10^−8^), and aged above 40 (*p* value = 2.18 × 10^−61^) have significantly more severe forehead wrinkles than age-matched females (Fig. 6a). Wrinkle severity shows a trend that is strongly dependent on age in both males and females. The proportion of males with forehead wrinkles experiences a persistent stepwise increase from 48% in males aged 18 − 20, to 59% in males aged 21 − 25, 59% in males aged 26 − 30, 72% in males aged 31 − 35, 79% in males aged 36 − 40, and 93% in males aged above 40 (chi-square test for trend *p* value < 0.001) (Fig. 6a). The proportion of females with forehead wrinkles also experiences a persistent stepwise increase from 33% in females aged 18 − 20, to 37% in females aged 21 − 25, 44% in females aged 26 − 30, 53% in females aged 31 − 35, 75% in females aged 36 − 40, and 94% in females aged above 40 (chi-square test for trend *p* value < 0.001) (Fig. 6a). Our results cross-validated the available literature on how forehead wrinkles are more severe in males below the age of 65 as compared to age-matched females.

A different study reported that Japanese women below the age of 30 develop pigment spots [9] and a Korean study observed that hyperpigmented macules are the main form of pigmentary changes with age in women [7]. We report that the ascertainment of pigment spots through self-reported means also returns the same trend in our SMCGES cohort. Overall, 16% of Chinese females in our current assessment report having pigment spots while just 13% of Chinese males report the same (*p* value = 3.47 × 10^−3^) (Fig. 6b). Next, to align our comparison with the available literature, we focused on age-matched comparisons for age groups below the age of 30. Significantly more females aged 18 − 20 (*p* value = 9.82 × 10^−6^), aged 21 − 25 (*p* value = 3.92 × 10^−1^), and aged 26 − 30 (*p* value = 5.13 × 10^−4^) have pigment spots than age-matched males (Fig. 6b). 24% of females aged 18 − 20 have pigment spots while just 19% of males report the same. We observe the same trend for participants aged 21 − 25–16% of females aged 21 − 25 have pigment spots while just 14% of males report the same. Lastly, 11% of females aged 26 − 30 and 9% of males aged 26 − 30 have pigment spots (Fig. 6b). Our results cross validated the available literature on how age-related pigmentary changes are prevalent in women below the age of 30, more so than in men.

All in all, our data demonstrates that the ascertainment of skin ageing phenotypes through self-reported means returns age- and sex-specific trends that are consistent with existing literature. More importantly, our results indicate that while our Spearman correlation values are weak, they do not hamper our ability to use self-reported phenotypic data to attain meaningful insights.

### Understanding and interpreting our results in the context of a sunny tropical environment

The participants in our SMCGES cohort are recruited and assessed in Singapore and Malaysia, both of which are sunny tropical countries. Based on Singapore’s National Environmental Agency [16], the Ultraviolet Index is graded as ‘Extreme’ from 1 to 3 pm on a typical day. Consequentially, participants from both countries are regularly exposed to large amounts of ultraviolet (UV) radiation. UV radiation is a well-studied risk factor for skin ageing. Hence, the skin ageing results from the SMCGES cohort should be interpreted in the context of an environment with high UV radiation exposure.

Meanwhile, as a result of evolutionary adaptation to environments with different amounts of UV radiation, some variation exists between the skin of people of different ethnicities. The effect of skin variation in other races (e.g., Malays and Indians) will be analysed when the number of participants of these races grows sufficiently large through ongoing efforts to expand the SMCGES cohort.

Despite analysing just the ethnic Chinese participants, we still observe a wide diversity in skin types. Skin type is measured using the Fitzpatrick Skin Type classification system. This is a classification system on whether the skin burns or tans in response to prolonged sun exposure. Participants with Fitzpatrick Skin Types III, IV, V, or VI are better protected from ultraviolet radiation from the Sun than participants with Fitzpatrick Skin Types I or II [10].

We wondered whether the six skin ageing scales studied in this paper considered the effects of skin type when designing their respective photo-numeric scales. Using Crow’s Feet wrinkles as an example, the proportion of participants with no Crow’s Feet wrinkles (i.e., photo 0 on the photo-numeric scale) and having Fitzpatrick Skin Types I through VI are treated as the reference group. Participants with few Crow’s Feet wrinkles (i.e., photo 1) on the photo-numeric scale have a significantly different composition of Fitzpatrick Skin Types as compared to the reference group (chi-square *p* value = 1.95 × 10^−5^). Similar comparisons were made between the reference group and participants with moderate Crow’s Feet wrinkles (i.e., photo 2) (chi-square *p* value = 3.98 × 10^−4^), severe Crow’s Feet wrinkles (i.e., photo 3) (chi-square *p* value = 2.02 × 10^−3^), and very severe Crow’s Feet wrinkles (i.e., photo 4) (chi-square *p* value = 2.33 × 10^−3^) (Fig. 7a).

Similar observations were made using the scale for forehead wrinkles. The proportion of participants with no forehead wrinkles (i.e., photo 0) and Fitzpatrick Skin Types I through VI are treated as the reference group. Statistically significant comparisons were observed between the reference group and participants with few forehead wrinkles (i.e., photo 1) (chi-square *p* value = 3.02 × 10^−4^), moderate forehead wrinkles (i.e., photo 2) (chi-square *p* value = 4.31 × 10^−10^), severe forehead wrinkles (i.e., photo 3) (chi-square *p* value = 9.47 × 10^−9^), and very severe forehead wrinkles (i.e., photo 4) (chi-square *p* value = 1.48 × 10^−5^) (Fig. 7b).

Evaluations for glabellar frowns returned similar results. The proportion of participants with no glabellar frowns (i.e., photo 0) and Fitzpatrick Skin Types I through VI are treated as the reference group. Statistically significant comparisons were observed between the reference group and participants with few glabellar frowns (i.e., photo 1) (chi-square *p* value = 2.25 × 10^−6^), moderate glabellar frowns (i.e., photo 2) (chi-square *p* value = 1.30 × 10^−5^), severe glabellar frowns (i.e., photo 3) (chi-square *p* value = 4.86 × 10^−4^), and very severe glabellar frowns (i.e., photo 4) (chi-square *p* value = 2.67 × 10^−4^) (Fig. 7c).

Similar results emerged from evaluations for nasolabial folds. The proportion of participants with no nasolabial folds (i.e., photo 0) and Fitzpatrick Skin Types I through VI are treated as the reference group. Statistically significant comparisons were observed between the reference group and participants with few nasolabial folds (i.e., photo 1) (chi-square *p* value = 4.92 × 10^−5^), moderate nasolabial folds (i.e., photo 2) (chi-square *p* value = 1.06 × 10^−6^), severe nasolabial folds (i.e., photo 3) (chi-square *p* value = 1.02 × 10^−9^), and very severe nasolabial folds (i.e., photo 4) (chi-square *p* value = 4.10 × 10^−10^) (Fig. 7d).

Next, for the wrinkling constituent of photo-ageing, we treat participants with grade 1 photo-ageing on the photo-ageing scale and Fitzpatrick Skin Types I through VI as the reference group. Statistically significant comparisons were observed between the reference group and participants with grade 2 photo-ageing (chi-square *p* value = 3.59 × 10^−7^), grade 3 photo-ageing (chi-square *p* value = 5.68 × 10^−11^), grade 4 photo-ageing (chi-square *p* value = 4.06 × 10^−4^), grade 5 photo-ageing (chi-square *p* value = 3.94 × 10^−3^), and grade 6 photo-ageing (chi-square *p* value = 3.48 × 10^−4^) (Fig. 7e).

Lastly, when the dyspigmentation constituent of photo-ageing is evaluated, similar results also emerge. The proportion of participants with photo 0 photo-ageing on this photo-ageing scale and Fitzpatrick Skin Types I through VI are treated as the reference group. Statistically significant comparisons were observed between the reference group and participants with photo 1 photo-ageing (chi-square *p* value = 1.01 × 10^−6^), photo 2 photo-ageing (chi-square *p* value = 3.76 × 10^−11^), photo 3 photo-ageing (chi-square *p* value = 8.24 × 10^−7^), photo 4 photo-ageing (chi-square *p* value = 5.11 × 10^−10^), and photo 5 photo-ageing (chi-square *p* value = 1.55 × 10^−5^) (Fig. 7f).

Our data shows that each of the six skin ageing scales studied in this paper has incorporated key elements of the six Fitzpatrick Skin Types in their respective scale design process. This is because the proportion of Fitzpatrick Skin Types I through VI in one photo-numeric grade is significantly different from the proportion of Fitzpatrick Skin Types I through VI in another photo-numeric grade. Thus, the fact that the participants from the SMCGES cohort have differences in skin variation does not appear to complicate the ability of the six scales studied in this paper to grade wrinkling and photo-ageing effectively.

### The effect of skin variation on other skin ageing phenotypes

Using the same method, we also studied 24 other skin ageing phenotypes using either photo-numeric scales or photographs (Table S1). We wondered whether the other skin ageing scales also considered the effects of skin type when designing their respective scale. We found that the photo-numeric scales for eleven other skin ageing phenotypes have also incorporated key elements of the six Fitzpatrick Skin Types in their respective scale design process. These eleven phenotypes can be broadly categorised based on their anatomical locations: ageing-related changes to the skin around the cheek (Fig. 8a–c), the eyes (Fig. 8d–g), and the lower face (Fig. 8h–k). The 13 remaining phenotypes should be thoroughly evaluated again with scales designed to better capture key elements of the six Fitzpatrick Skin Types (Figures S1i–S1xiii).

### Future work

Our observation that self-reported skin ageing data is comparable to the evaluations done by our trained assessors contributes to discussions in the field on the best ways to evaluate skin characteristics. A recent study found that self-reported skin colour using a photo-numeric scale is concordant with objective measurements [15]. Localised changes in skin pigmentation are one of the key hallmarks of ageing skin [17]. Furthermore, Chinese skin is known to become dyspigmented more easily with age [21]. We therefore see a potential future direction to validate self-reported skin dyspigmentation patterns using suitable photo-numeric scales. This future work can be conducted when more photo-numeric scales for dyspigmentation patterns are developed and properly validated.

The main weakness of this validation study is that while we have used a large sample size of 1081 ethnic Chinese participants, scaling this further up to our full dataset of 3365 participants is labour-intensive. As a result, it will be challenging to deliver any findings in a timely manner. Meanwhile, a meta-analysis published by our team [23] uncovered significant epidemiological risk factors (e.g., age, gender, ethnicity, air pollution, smoking, nutrition, and sun exposure) associated with skin ageing phenotypes. The ability to use self-reported skin ageing data enables us to scale up our findings in large-scale epidemiological studies, such as in a population of 3365 participants, in a reasonable time frame. This in turn enables us to explore the association between various risk factors and different forms of skin ageing in a larger population.

At present, there are a growing number of studies using self-reported skin data [3, 4, 8, 11, 13, 15, 19, 20]. Here, we showed that self-reported signs of skin ageing provide a satisfactory approximation to the signs of skin ageing identified by our assessors. Using self-reported signs of skin ageing improves the scalability of our findings in large-scale epidemiological studies. Future work will focus on investigating the associations between risk factors and a variety of skin ageing phenotypes.

## Conclusion

In conclusion, our study contributes to ongoing discussions on different methods to evaluate skin ageing. We examined the suitability of using self-reported skin ageing data and compared it to skin ageing assessments evaluated by our trained assessors. Here, we demonstrate that self-reported data on wrinkling and photo-ageing is concordant with assessments conducted by our trained assessors. The same skin is consistently evaluated to have the same severity grade on the same photo-numeric scale regardless of whether the participant or one of the assessors is making the assessment. These findings underscore the reliability and validity of self-reported skin ageing data, providing a valuable tool for large-scale epidemiological studies. Our future work will focus on exploring the associations between risk factors and different forms of skin ageing to advance our understanding of this complex ageing phenomenon.

### Supplementary Information

Additional file 1: Figure S1i: Distribution of Fitzpatrick Skin Types I to VI across participants with different appearance of cheek pores. Whether cheek pores appear larger is quantified as a binary grade. Photos of large cheek pores are obtained from Volume 2 of the Skin Ageing Atlas. Participants are deemed to have cheek pores that appear larger if their skin cheek pores appear similar to the photos from Volume 2 of the Skin Ageing Atlas. The Chi-square test p-values for all the pair-wise comparisons conducted were corrected for multiple testing through the Bonferroni Correction method. The citation for Volume 2 of the Skin Ageing Atlas, in which photos used for assessing whether cheek pores appear larger are available, can be found in Supplementary Table S1. Figure S1ii: Distribution of Fitzpatrick Skin Types I to VI across participants with different severities of cheek folds. Cheek folds are quantified as a grade on a validated photonumeric scale. The Chi-square test p-values for all the pair-wise comparisons conducted were corrected for multiple testing through the Bonferroni Correction method. The photonumeric scale used for this phenotypic assessment can be found in Supplementary Table S1. Figure S1iii: Distribution of Fitzpatrick Skin Types I to VI across participants with or without permanent erythema. Permanent erythema is quantified as a binary grade. Photos of permanent erythema are obtained from Volume 2 of Dermatology (Third Edition). Participants are deemed to have permanent erythema if the skin of their cheeks appears similar to the photos from Volume 2 of Dermatology (Third Edition). The Chi-square test p-values for all the pair-wise comparisons conducted were corrected for multiple testing through the Bonferroni Correction method. The citation for Volume 2 of Dermatology (Third Edition), in which photos used for assessing permanent erythema are available, can be found in Supplementary Table S1. Figure S1iv: Distribution of Fitzpatrick Skin Types I to VI across participants with different severities of telangiectasias. Telangiectasias are quantified as a grade on a validated photo-numeric scale. The Chi-square test p-values for all the pair-wise comparisons conducted were corrected for multiple testing through the Bonferroni Correction method. The photo-numeric scale used for this phenotypic assessment can be found in Supplementary Table S1. Figure S1v: Distribution of Fitzpatrick Skin Types I to VI across participants with or without horizontal interocular wrinkles. Horizontal interocular wrinkles are quantified as a binary grade. Photos of horizontal interocular wrinkles are obtained from Volume 2 of the Skin Ageing Atlas. Participants are deemed to have horizontal interocular wrinkles if their skin appear similar to the photos from Volume 2 of the Skin Ageing Atlas. The Chi-square test p-values for all the pair-wise comparisons conducted were corrected for multiple testing through the Bonferroni Correction method. The citation for Volume 2 of the Skin Ageing Atlas, in which photos used for assessing horizontal interocular wrinkles are available, can be found in Supplementary Table S1. Figure S1vi: Distribution of Fitzpatrick Skin Types I to VI across participants with different fullness of the upper lip. Upper lip fullness is quantified as a grade on a validated photonumeric scale. The Chi-square test p-values for all the pair-wise comparisons conducted were corrected for multiple testing through the Bonferroni Correction method. The photonumeric scale used for this phenotypic assessment can be found in Supplementary Table S1. Figure S1vii: Distribution of Fitzpatrick Skin Types I to VI across participants with different fullness of the lower lip. Lower lip fullness is quantified as a grade on a validated photonumeric scale. The Chi-square test p-values for all the pair-wise comparisons conducted were corrected for multiple testing through the Bonferroni Correction method. The photonumeric scale used for this phenotypic assessment can be found in Supplementary Table S1. Figure S1viii: Distribution of Fitzpatrick Skin Types I to VI across participants with or without milia. Milia are quantified as a binary grade. Photos of milia are obtained from Skin Disease Diagnosis and Treatment. Participants are deemed to have milia if their skin appear similar to the photos from Skin Disease Diagnosis and Treatment. The Chi-square test p-values for all the pair-wise comparisons conducted were corrected for multiple testing through the Bonferroni Correction method. The citation for Skin Disease Diagnosis and Treatment, in which photos used for assessing milia are available, can be found in Supplementary Table S1. Figure S1ix: Distribution of Fitzpatrick Skin Types I to VI across participants with or without freckles. Freckles are quantified as a binary grade. Photos of freckles are obtained from Ferri's Fast Facts in Dermatology. Participants are deemed to have freckles if their skin appear similar to the photos from Ferri's Fast Facts in Dermatology. The Chi-square test p-values for all the pair-wise comparisons conducted were corrected for multiple testing through the Bonferroni Correction method. The citation for Ferri's Fast Facts in Dermatology, in which photos used for assessing freckles are available, can be found in Supplementary Table S1. Figure S1x: Distribution of Fitzpatrick Skin Types I to VI across participants with or without solar comedones. Solar comedones are quantified as a binary grade. Photos of solar comedones are obtained from Roxburgh's Common Skin Diseases and Skin Disease Diagnosis and Treatment. Participants are deemed to have solar comedones if their skin appear similar to the photos from Roxburgh's Common Skin Diseases and Skin Disease Diagnosis and Treatment. The Chi-square test p-values for all the pair-wise comparisons conducted were corrected for multiple testing through the Bonferroni Correction method. The citations for Roxburgh's Common Skin Diseases and Skin Disease Diagnosis and Treatment, in which photos used for assessing solar comedones are available, can be found in Supplementary Table S1. Figure S1xi: Distribution of Fitzpatrick Skin Types I to VI across participants with or without uneven skin pigmentation. Uneven skin pigmentation is quantified as a binary grade. Photos of uneven skin pigmentation are obtained from Ferri's Fast Facts in Dermatology. Participants are deemed to have uneven skin pigmentation if their skin appear similar to the photos from Ferri's Fast Facts in Dermatology. The Chi-square test p-values for all the pair-wise comparisons conducted were corrected for multiple testing through the Bonferroni Correction method. The citation for Ferri's Fast Facts in Dermatology, in which photos used for assessing uneven skin pigmentation are available, can be found in Supplementary Table S1. Figure S1xii: Distribution of Fitzpatrick Skin Types I to VI across participants with or without sebaceous hyperplasia. Sebaceous hyperplasia is quantified as a binary grade. Photos of sebaceous hyperplasia are obtained from Diagnosis of Aging Skin Diseases, Roxburgh's Common Skin Diseases, and Skin Disease Diagnosis and Treatment. Participants are deemed to have sebaceous hyperplasia if their skin appear similar to the photos from Diagnosis of Aging Skin Diseases, Roxburgh's Common Skin Diseases, and Skin Disease Diagnosis and Treatment. The Chi-square test p-values for all the pair-wise comparisons conducted were corrected for multiple testing through the Bonferroni Correction method. The citations for Diagnosis of Aging Skin Diseases, Roxburgh's Common Skin Diseases, and Skin Disease Diagnosis and Treatment, in which photos used for assessing sebaceous hyperplasia are available, can be found in Supplementary Table S1. Figure S1xiii: Distribution of Fitzpatrick Skin Types I to VI across participants with or without seborrheic keratosis. Seborrheic keratosis is quantified as a binary grade. Photos of seborrheic keratosis are obtained from Volume 2 of Dermatology (Third Edition), Handbook of Skin Diseases, and Skin Disease Diagnosis and Treatment. Participants are deemed to have seborrheic keratosis if their skin appear similar to the photos from Volume 2 of Dermatology (Third Edition), Handbook of Skin Diseases, and Skin Disease Diagnosis and Treatment. The Chi-square test p-values for all the pair-wise comparisons conducted were corrected for multiple testing through the Bonferroni Correction method. The citations for Volume 2 of Dermatology (Third Edition), Handbook of Skin Diseases, and Skin Disease Diagnosis and Treatment, in which photos used for assessing seborrheic keratosis are available, can be found in Supplementary Table S1. Table S1: Sources of the validated photo-numeric scales. Table S2: Comparison between skin ageing phenotypes grades by assessors and participants evaluated using Spearman's Rank Correlation, Cohen's Kappa, sensitivity, and specificity.

## Data Availability

All data used and included in this study are available from the corresponding author (F.T.C.).

## Abbreviations

SMCGES: Singapore/Malaysia Cross-section Genetics Study
ISAAC: International Study of Asthma and Allergies in Childhood
IBM SPSS/PC: International Business Machines Corporation Statistical Package for Social Scientists Personal Computer (SPSS/PC)
EOS: Electro-Optical System
IS: Image stabilisation
USM: Ultrasonic motor
EF: Electronic focus
SD: Standard deviation
SGD: Singapore dollars
HDB (Public housing): Public housing constructed by the Singapore Housing Development Board
BMI: Body mass index
cm: Centimetres
m^2^: Squared metres
kg: Kilograms
ROC: Receiver operator characteristic
AUC: Area under the curve
