# Supplementary material for: Comparisons between wrinkles and photo-ageing detected and self-reported by the participant or identified by trained assessors reveal insights from Chinese individuals in the Singapore/Malaysia Cross-sectional Genetics Epidemiology Study (SMCGES) cohort
Source: J Physiol Anthropol. 2024 May 18;43:14. doi: 10.1186/s40101-024-00361-8 (PMC11102249; doi:10.1186/s40101-024-00361-8)
Supplement: Supplementary file 1 — Additional file 1: Figure S1i: Distribution of Fitzpatrick Skin Types I to VI across participants with different appearance of cheek pores. Whether cheek pores appear larger is quantified as a binary grade. Photos of large cheek pores are obtained from Volume 2 of the Skin Ageing Atlas. Participants are deemed to have cheek pores that appear larger if their skin cheek pores appear similar to the photos from Volume 2 of the Skin Ageing Atlas. The Chi-square test p-values for all the pair-wise comparisons conducted were corrected for multiple testing through the Bonferroni Correction method. The citation for Volume 2 of the Skin Ageing Atlas, in which photos used for assessing whether cheek pores appear larger are available, can be found in Supplementary Table S1. Figure S1ii: Distribution of Fitzpatrick Skin Types I to VI across participants with different severities of cheek folds. Cheek folds are quantified as a grade on a validated photonumeric scale. The Chi-square test p-values for all the pair-wise comparisons conducted were corrected for multiple testing through the Bonferroni Correction method. The photonumeric scale used for this phenotypic assessment can be found in Supplementary Table S1. Figure S1iii: Distribution of Fitzpatrick Skin Types I to VI across participants with or without permanent erythema. Permanent erythema is quantified as a binary grade. Photos of permanent erythema are obtained from Volume 2 of Dermatology (Third Edition). Participants are deemed to have permanent erythema if the skin of their cheeks appears similar to the photos from Volume 2 of Dermatology (Third Edition). The Chi-square test p-values for all the pair-wise comparisons conducted were corrected for multiple testing through the Bonferroni Correction method. The citation for Volume 2 of Dermatology (Third Edition), in which photos used for assessing permanent erythema are available, can be found in Supplementary Table S1. Figure S1iv: Distribution of Fitzpatrick Skin Types [file 40101_2024_361_MOESM1_ESM.zip › Individual Figures S1.pdf]

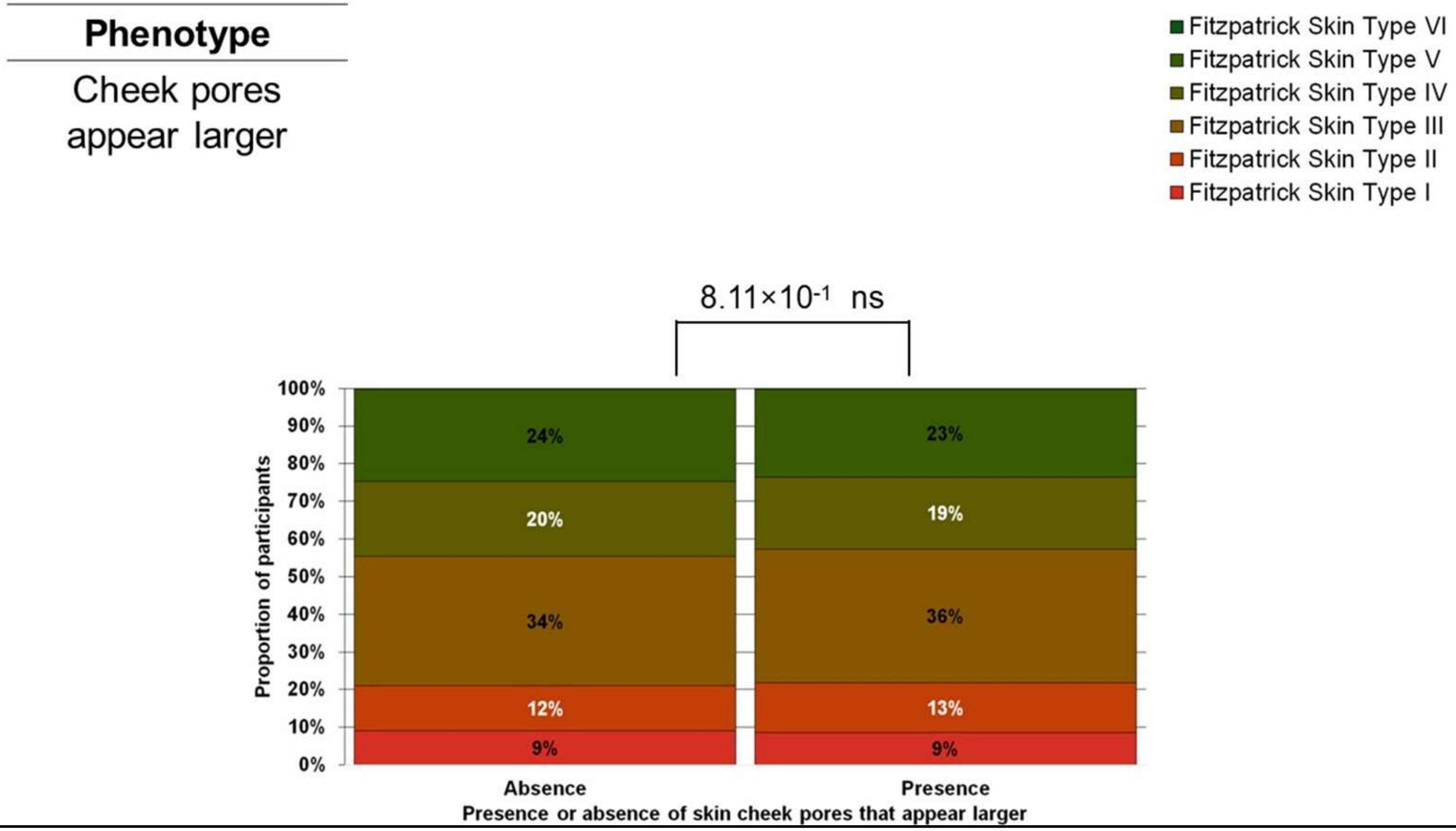

Figure S1i: Distribution of Fitzpatrick Skin Types I to VI across participants with different appearance of cheek pores. Whether cheek pores appear larger is quantified as a binary grade. Photos of large cheek pores are obtained from Volume 2 of the Skin Ageing Atlas. Participants are deemed to have cheek pores that appear larger if their skin cheek pores appear similar to the photos from Volume 2 of the Skin Ageing Atlas. The Chi-square test p-values for all the pair-wise comparisons conducted were corrected for multiple testing through the Bonferroni Correction method. The citation for Volume 2 of the Skin Ageing Atlas, in which photos used for assessing whether cheek pores appear larger are available, can be found in Supplementary Table S1.

Phenotype
Cheek folds

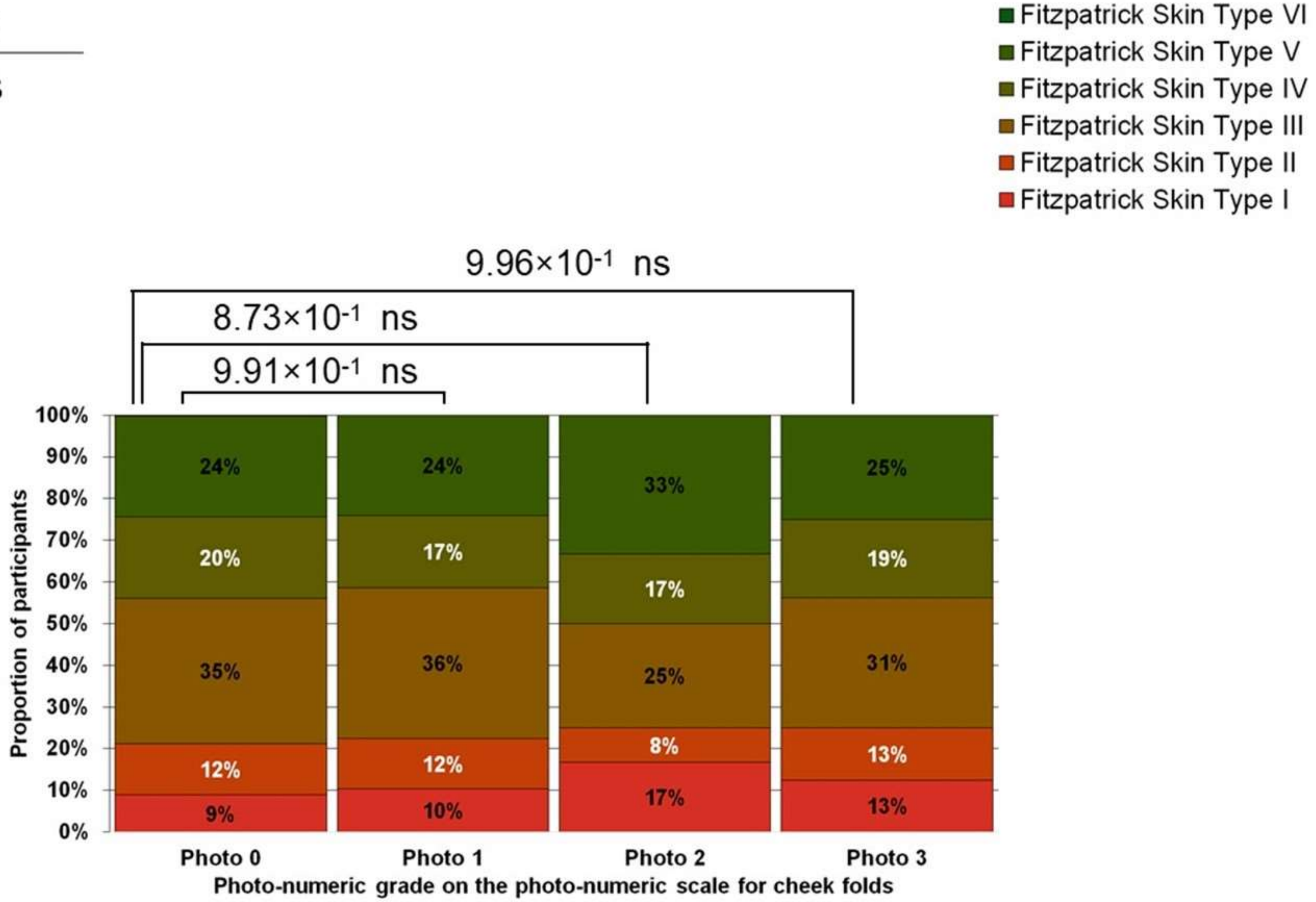

Figure S1ii: Distribution of Fitzpatrick Skin Types I to VI across participants with different severities of cheek folds. Cheek folds are quantified as a grade on a validated photo-numeric scale. The Chi-square test p-values for all the pair-wise comparisons conducted were corrected for multiple testing through the Bonferroni Correction method. The photo-numeric scale used for this phenotypic assessment can be found in Supplementary Table S1.

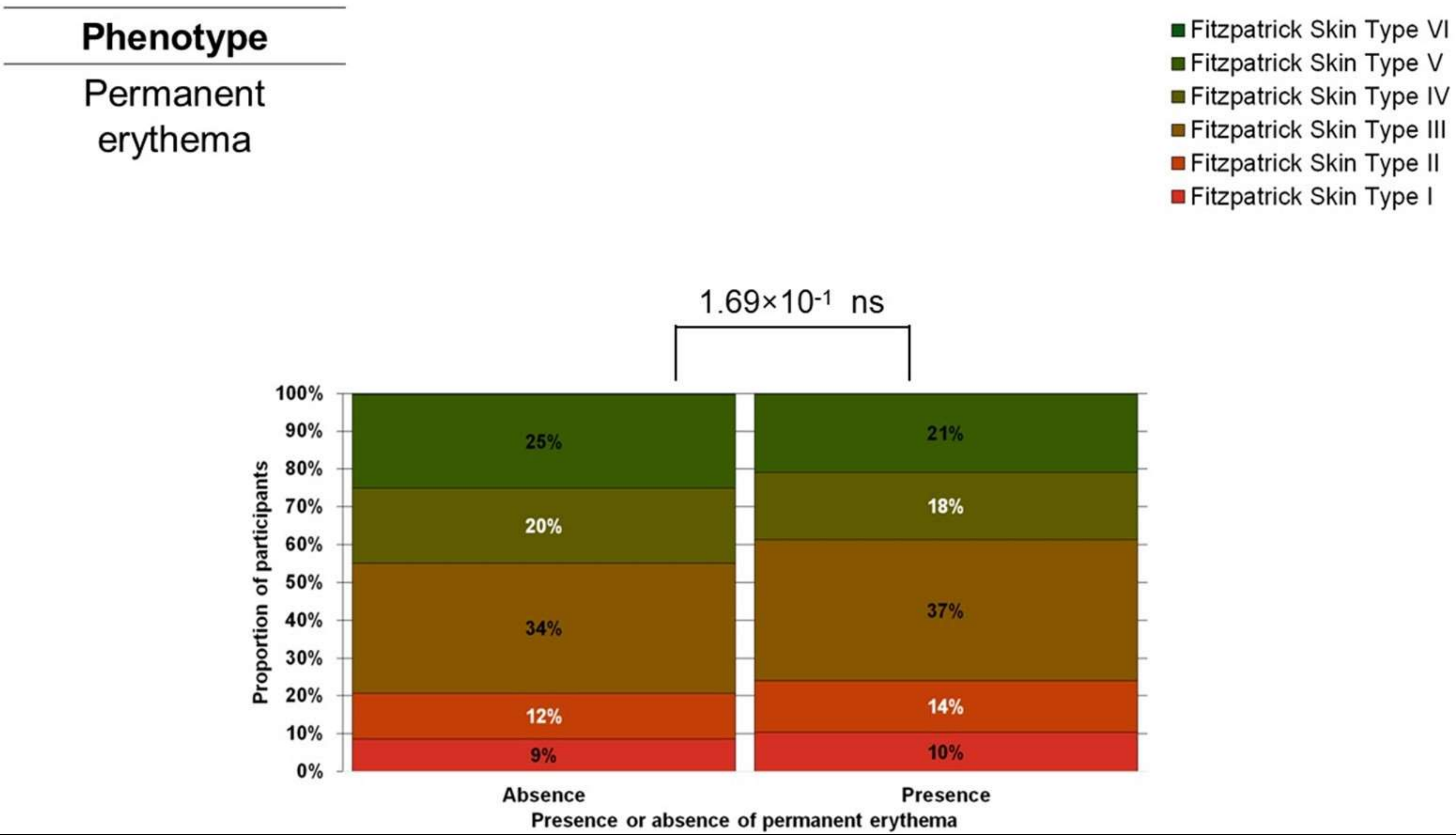

Figure S1iii: Distribution of Fitzpatrick Skin Types I to VI across participants with or without permanent erythema. Permanent erythema is quantified as a binary grade. Photos of permanent erythema are obtained from Volume 2 of Dermatology (Third Edition). Participants are deemed to have permanent erythema if the skin of their cheeks appears similar to the photos from Volume 2 of Dermatology (Third Edition). The Chi-square test p-values for all the pair-wise comparisons conducted were corrected for multiple testing through the Bonferroni Correction method. The citation for Volume 2 of Dermatology (Third Edition), in which photos used for assessing permanent erythema are available, can be found in Supplementary Table S1.

Phenotype
Telangiectasias

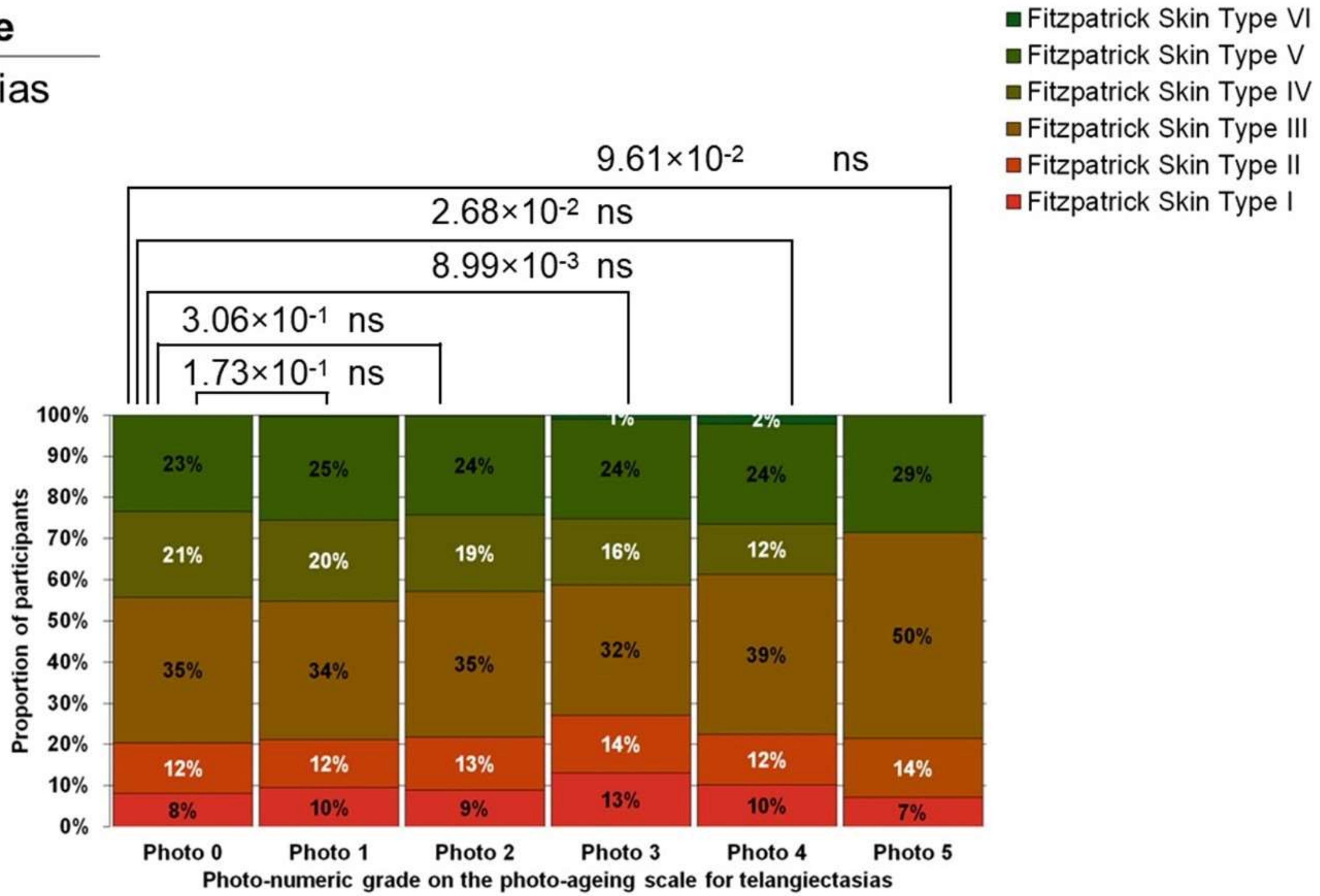

Figure S1iv: Distribution of Fitzpatrick Skin Types I to VI across participants with different severities of telangiectasias. Telangiectasias are quantified as a grade on a validated photo-numeric scale. The Chi-square test p-values for all the pair-wise comparisons conducted were corrected for multiple testing through the Bonferroni Correction method. The photo-numeric scale used for this phenotypic assessment can be found in Supplementary Table S1.

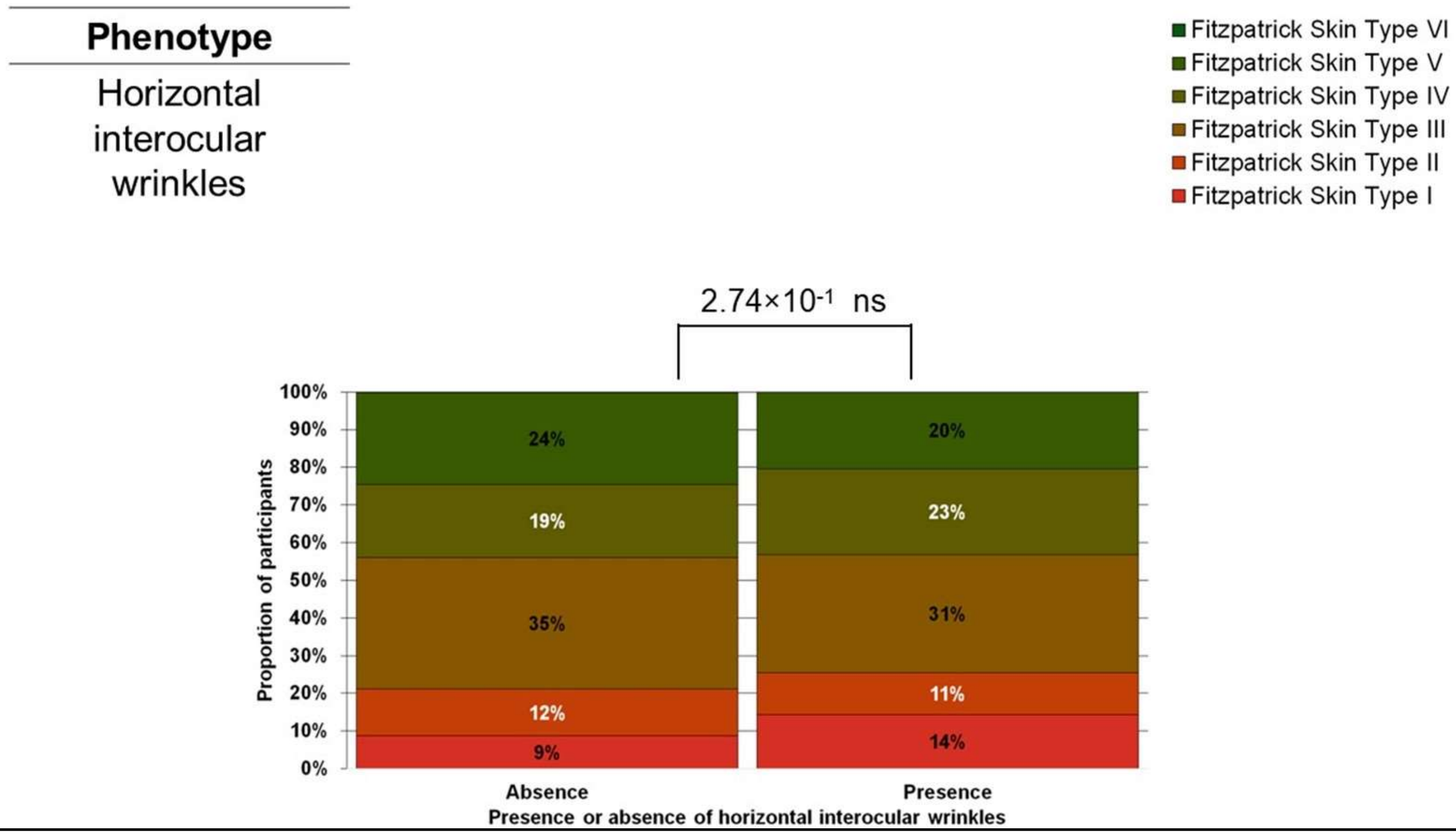

Figure S1v: Distribution of Fitzpatrick Skin Types I to VI across participants with or without horizontal interocular wrinkles. Horizontal interocular wrinkles are quantified as a binary grade. Photos of horizontal interocular wrinkles are obtained from Volume 2 of the Skin Ageing Atlas. Participants are deemed to have horizontal interocular wrinkles if their skin appear similar to the photos from Volume 2 of the Skin Ageing Atlas. The Chi-square test p-values for all the pair-wise comparisons conducted were corrected for multiple testing through the Bonferroni Correction method. The citation for Volume 2 of the Skin Ageing Atlas, in which photos used for assessing horizontal interocular wrinkles are available, can be found in Supplementary Table S1.

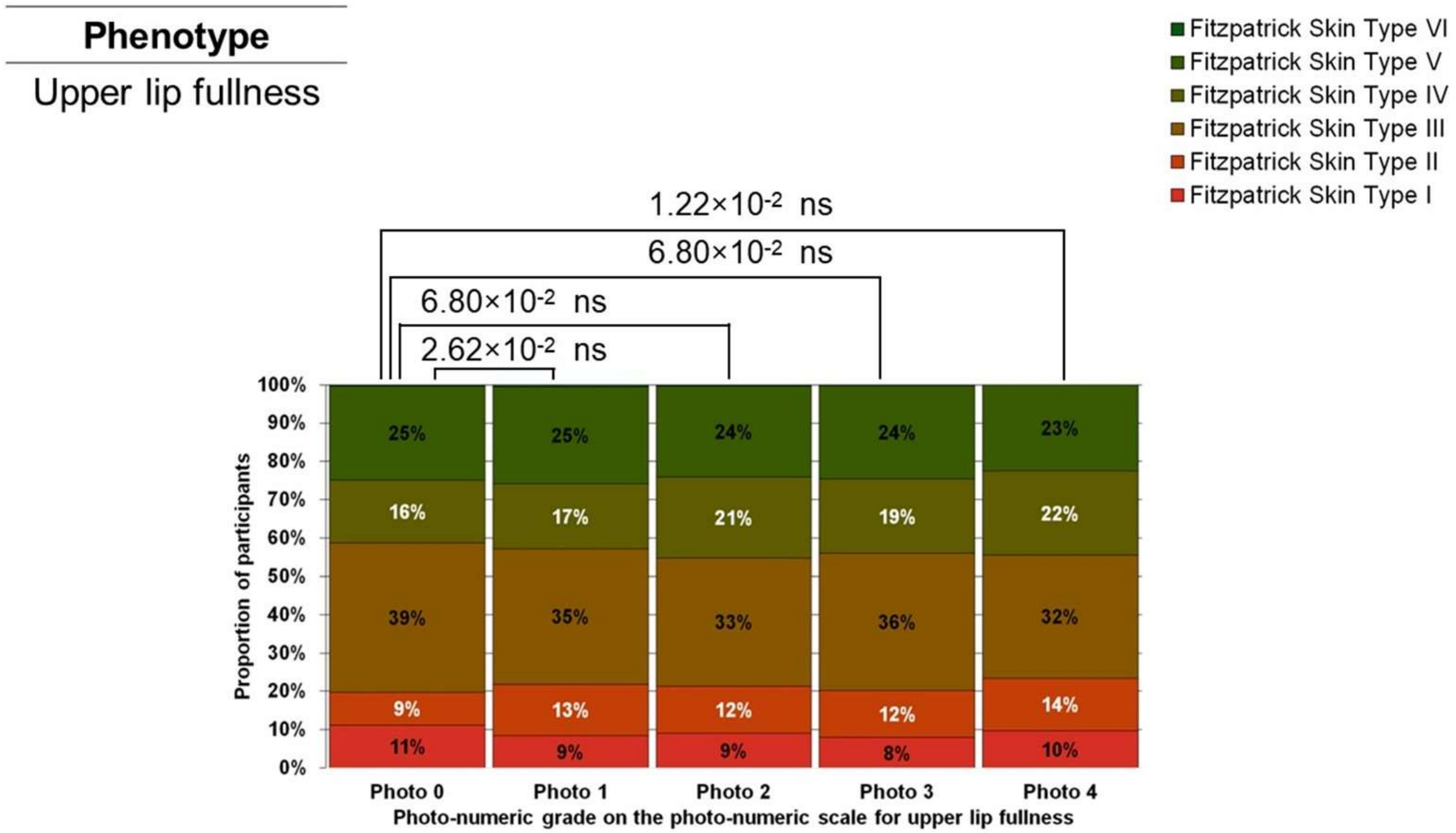

Figure S1vi: Distribution of Fitzpatrick Skin Types I to VI across participants with different fullness of the upper lip. Upper lip fullness is quantified as a grade on a validated photo-numeric scale. The Chi-square test p-values for all the pair-wise comparisons conducted were corrected for multiple testing through the Bonferroni Correction method. The photo-numeric scale used for this phenotypic assessment can be found in Supplementary Table S1.

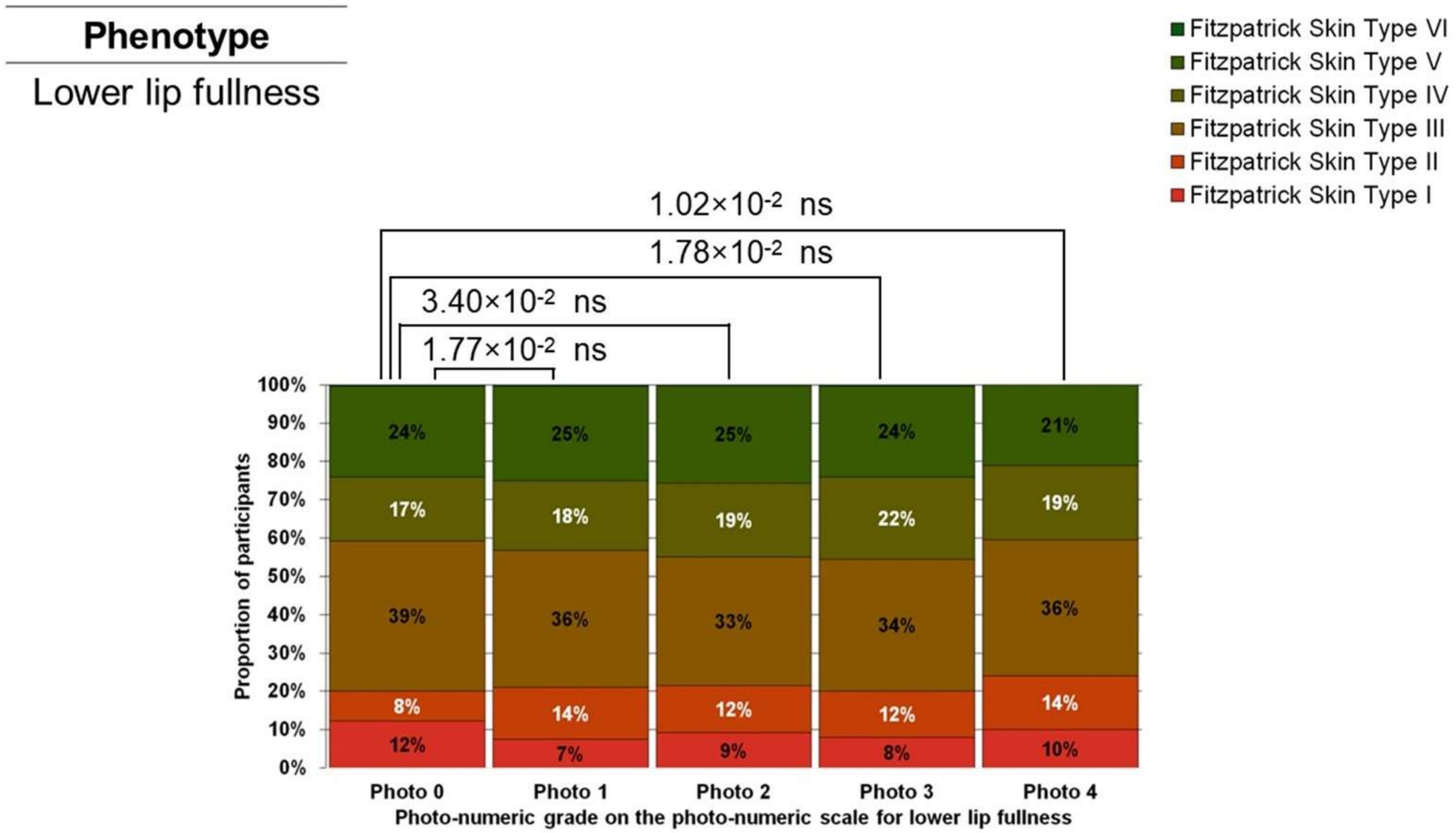

Figure S1vii: Distribution of Fitzpatrick Skin Types I to VI across participants with different fullness of the lower lip. Lower lip fullness is quantified as a grade on a validated photo-numeric scale. The Chi-square test p-values for all the pair-wise comparisons conducted were corrected for multiple testing through the Bonferroni Correction method. The photo-numeric scale used for this phenotypic assessment can be found in Supplementary Table S1.

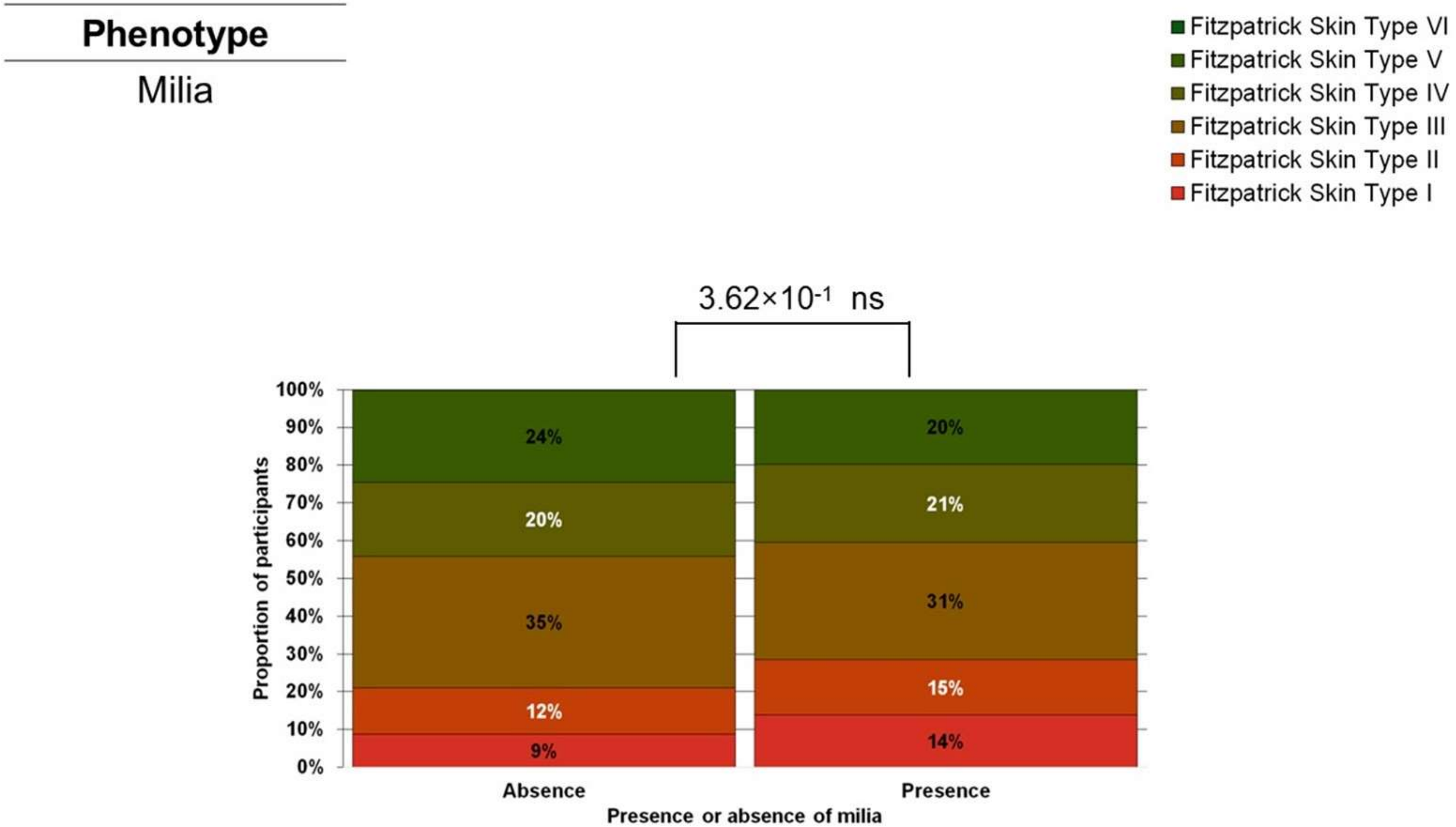

Figure S1viii: Distribution of Fitzpatrick Skin Types I to VI across participants with or without milia. Milia are quantified as a binary grade. Photos of milia are obtained from Skin Disease Diagnosis and Treatment. Participants are deemed to have milia if their skin appear similar to the photos from Skin Disease Diagnosis and Treatment. The Chi-square test p-values for all the pair-wise comparisons conducted were corrected for multiple testing through the Bonferroni Correction method. The citation for Skin Disease Diagnosis and Treatment, in which photos used for assessing milia are available, can be found in Supplementary Table S1.

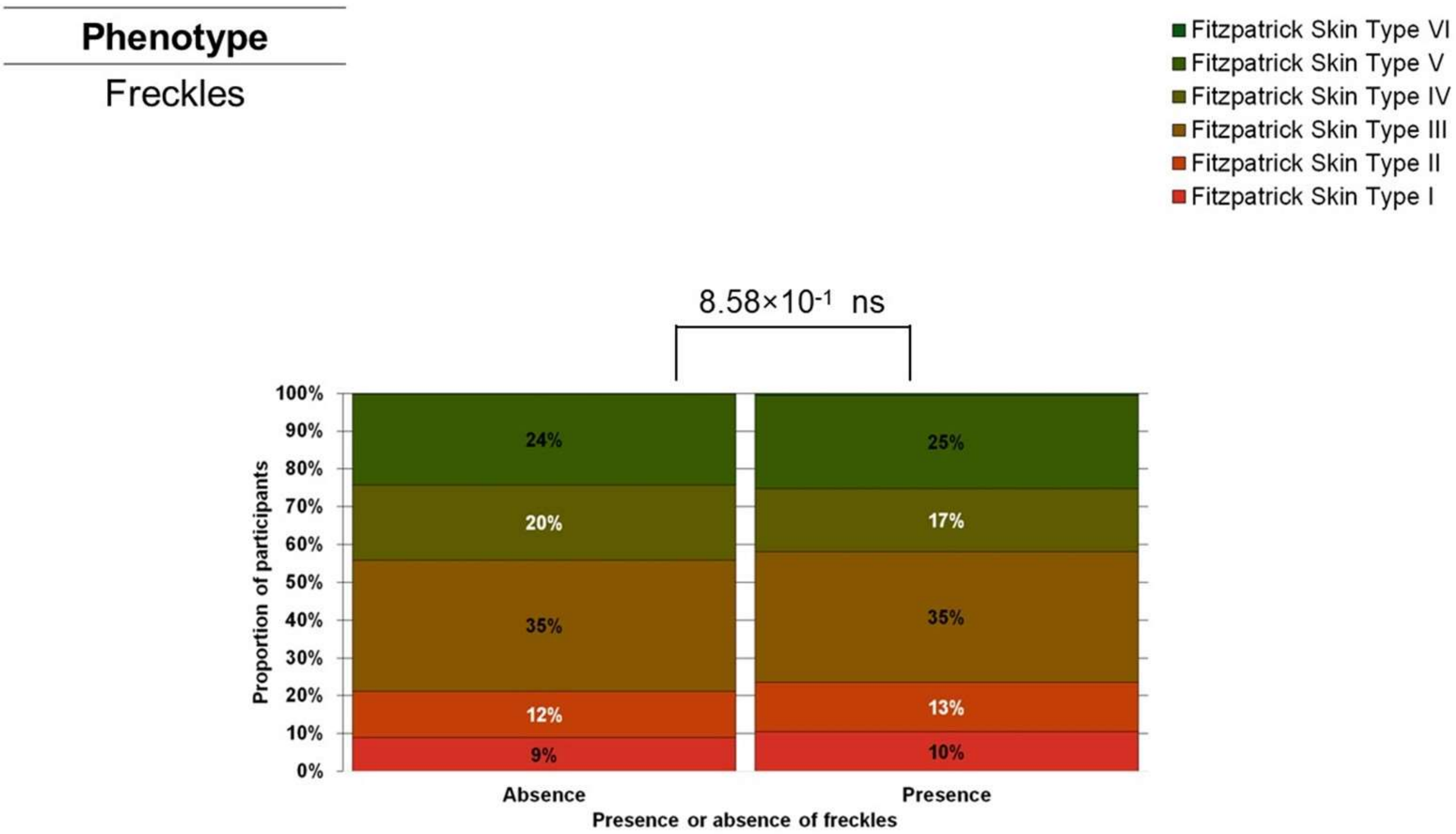

Figure S1ix: Distribution of Fitzpatrick Skin Types I to VI across participants with or without freckles. Freckles are quantified as a binary grade. Photos of freckles are obtained from Ferri's Fast Facts in Dermatology. Participants are deemed to have freckles if their skin appear similar to the photos from Ferri's Fast Facts in Dermatology. The Chi-square test p-values for all the pair-wise comparisons conducted were corrected for multiple testing through the Bonferroni Correction method. The citation for Ferri's Fast Facts in Dermatology, in which photos used for assessing freckles are available, can be found in Supplementary Table S1.

Phenotype
Solar comedone

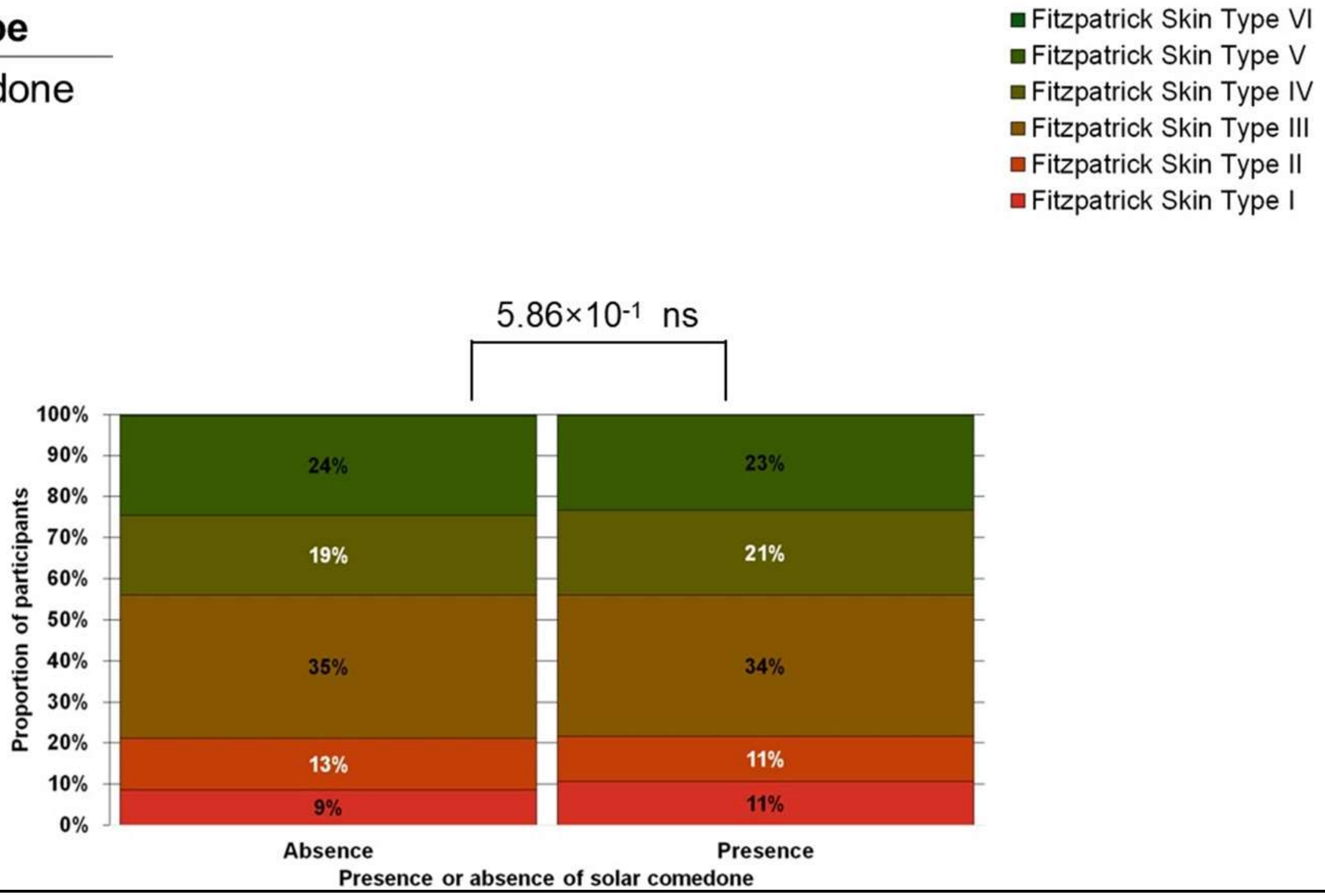

Figure S1x: Distribution of Fitzpatrick Skin Types I to VI across participants with or without solar comedones. Solar comedones are quantified as a binary grade. Photos of solar comedones are obtained from Roxburgh's Common Skin Diseases and Skin Disease Diagnosis and Treatment. Participants are deemed to have solar comedones if their skin appear similar to the photos from Roxburgh's Common Skin Diseases and Skin Disease Diagnosis and Treatment. The Chi-square test p-values for all the pair-wise comparisons conducted were corrected for multiple testing through the Bonferroni Correction method. The citations for Roxburgh's Common Skin Diseases and Skin Disease Diagnosis and Treatment, in which photos used for assessing solar comedones are available, can be found in Supplementary Table S1.

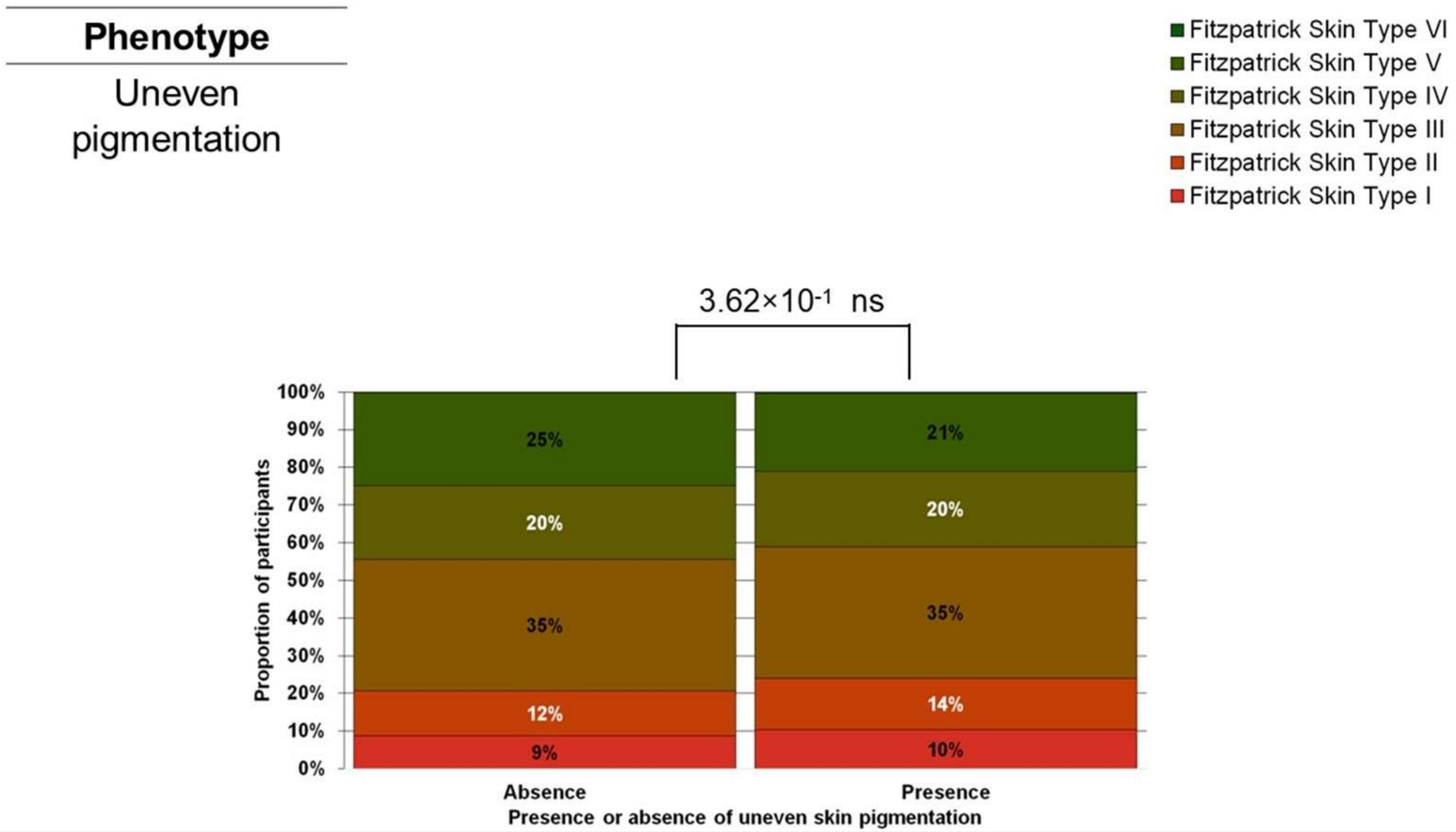

Figure S1xi: Distribution of Fitzpatrick Skin Types I to VI across participants with or without uneven skin pigmentation. Uneven skin pigmentation is quantified as a binary grade. Photos of uneven skin pigmentation are obtained from Ferri's Fast Facts in Dermatology. Participants are deemed to have uneven skin pigmentation if their skin appear similar to the photos from Ferri's Fast Facts in Dermatology. The Chi-square test p-values for all the pair-wise comparisons conducted were corrected for multiple testing through the Bonferroni Correction method. The citation for Ferri's Fast Facts in Dermatology, in which photos used for assessing uneven skin pigmentation are available, can be found in Supplementary Table S1.

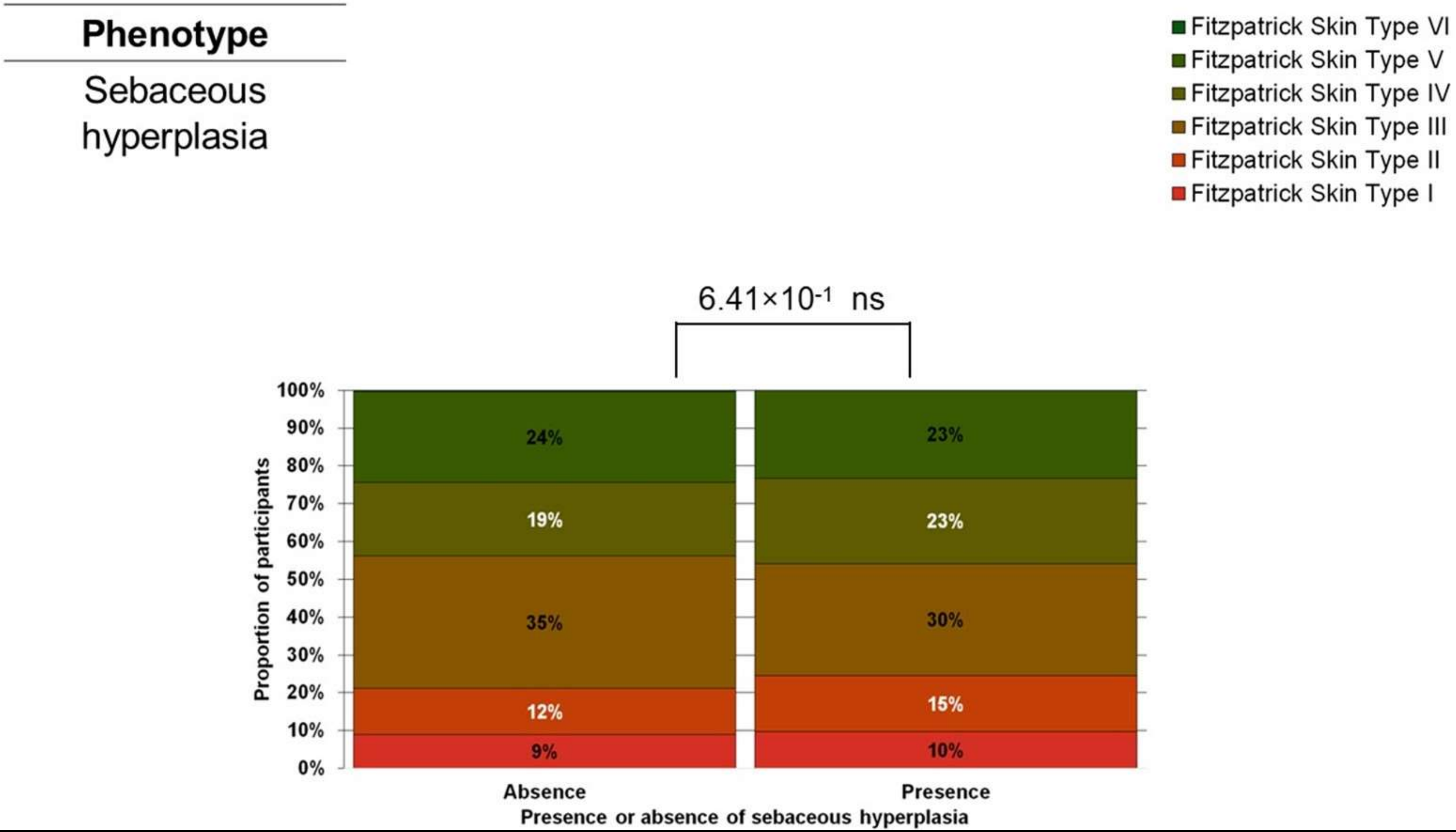

Figure S1xii: Distribution of Fitzpatrick Skin Types I to VI across participants with or without sebaceous hyperplasia. Sebaceous hyperplasia is quantified as a binary grade. Photos of sebaceous hyperplasia are obtained from Diagnosis of Aging Skin Diseases, Roxburgh's Common Skin Diseases, and Skin Disease Diagnosis and Treatment. Participants are deemed to have sebaceous hyperplasia if their skin appear similar to the photos from Diagnosis of Aging Skin Diseases, Roxburgh's Common Skin Diseases, and Skin Disease Diagnosis and Treatment. The Chi-square test p-values for all the pair-wise comparisons conducted were corrected for multiple testing through the Bonferroni Correction method. The citations for Diagnosis of Aging Skin Diseases, Roxburgh's Common Skin Diseases, and Skin Disease Diagnosis and Treatment, in which photos used for assessing sebaceous hyperplasia are available, can be found in Supplementary Table S1.

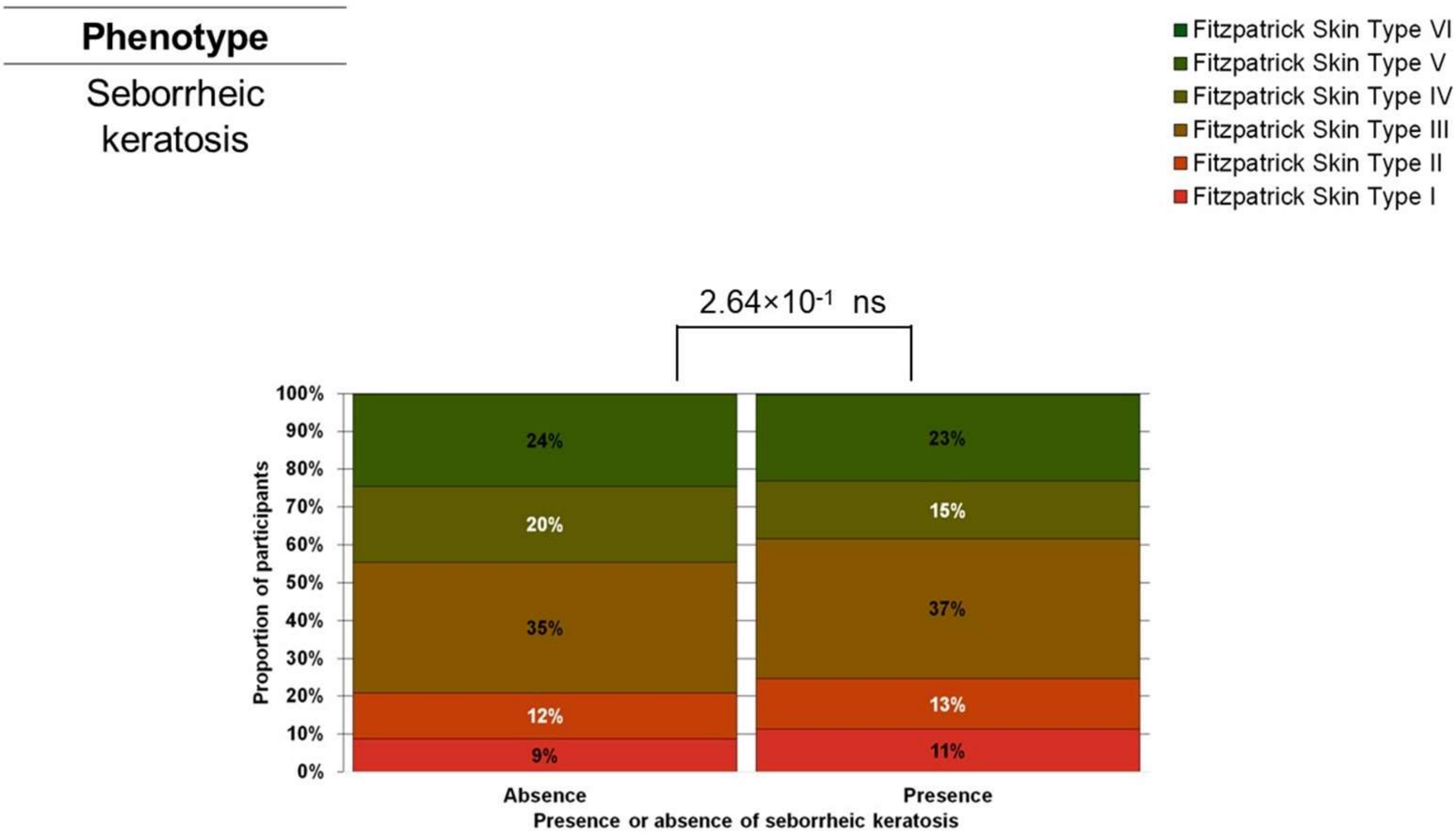

Figure S1xiii: Distribution of Fitzpatrick Skin Types I to VI across participants with or without seborrheic keratosis. Seborrheic keratosis is quantified as a binary grade. Photos of seborrheic keratosis are obtained from Volume 2 of Dermatology (Third Edition), Handbook of Skin Diseases, and Skin Disease Diagnosis and Treatment. Participants are deemed to have seborrheic keratosis if their skin appear similar to the photos from Volume 2 of Dermatology (Third Edition), Handbook of Skin Diseases, and Skin Disease Diagnosis and Treatment. The Chi-square test p-values for all the pair-wise comparisons conducted were corrected for multiple testing through the Bonferroni Correction method. The citations for Volume 2 of Dermatology (Third Edition), Handbook of Skin Diseases, and Skin Disease Diagnosis and Treatment, in which photos used for assessing seborrheic keratosis are available, can be found in Supplementary Table S1.
